# GLP-1 Receptor Agonist Therapy in Patients with Cancer: A Review of Clinical Outcomes

**DOI:** 10.3390/ijms27209251

**Published:** 2026-10-18

**Authors:** Alexandra Cătălina Andronescu, Lorena Ciumărnean, Olga Hilda Orășan, Angela Cozma, Nicoleta Valentina Leah, Roxana Liana Lucaciu, Adriana Corina Hangan, Lucia Maria Procopciuc

**Affiliations:** 1 2nd Department, Faculty of Nursing and Health Sciences, “Iuliu Hațieganu” University of Medicine and Pharmacy, 400015 Cluj-Napoca, Romania; andronescu_alexa_cata@elearn.umfcluj.ro (A.C.A.); nicoleta.leach@umfcluj.ro (N.V.L.); 2 Department of Internal Medicine, 4th Medical Discipline, “Iuliu Hațieganu” University of Medicine and Pharmacy, 400015 Cluj-Napoca, Romania; hilda.orasan@umfcluj.ro (O.H.O.); angelacozma@umfcluj.ro (A.C.); 3 Department of Pharmaceutical Biochemistry and Clinical Laboratory, Faculty of Pharmacy, “Iuliu Hațieganu” University of Medicine and Pharmacy, 400349 Cluj-Napoca, Romania; liana.lucaciu@umfcluj.ro; 4 Department of Inorganic Chemistry, Faculty of Pharmacy, “Iuliu Hațieganu” University of Medicine and Pharmacy, 400012 Cluj-Napoca, Romania; adriana.hangan@umfcluj.ro; 5 Department of Medical Biochemistry, Faculty of Medicine, “Iuliu Hațieganu” University of Medicine and Pharmacy, 400349 Cluj-Napoca, Romania; lprocopciuc@umfcluj.ro

**Keywords:** glucagon-like peptide-1 receptor agonist, cancer, overall survival, mortality, recurrence, disease progression

## Abstract

Glucagon-like peptide-1 receptor agonists (GLP-1 RAs) are increasingly used for the management of obesity and type 2 diabetes, including among patients with cancer. Although previous research has mainly focused on cancer incidence, their associations with clinical outcomes after cancer diagnosis remain less well characterized. Therefore, the present study aimed to evaluate the oncologic and survival outcomes associated with GLP-1 RA use in adults with an established malignancy. We performed a systematic review of comparative studies evaluating GLP-1 RA use in patients with a confirmed cancer diagnosis. PubMed, Scopus and Web of Science were searched for English-language publications from 1 January 2016 to 8 August 2026. Twenty-six retrospective cohort studies were included in the qualitative synthesis. Thirteen studies included selective GLP-1 RAs and tirzepatide within the same exposure group. Study quality was assessed using the Newcastle–Ottawa Scale. Twenty-five studies evaluated overall survival and/or all-cause mortality, while nine assessed cancer recurrence, progression, or related outcomes. Survival and mortality outcomes more often favored the exposed group (especially compared with nonuse) than not, but findings were less consistent in active-comparator analyses. Evidence for recurrence and progression was sparse and heterogeneous, and no clear signal of increased risk was observed. Because all of these were observational studies with substantial differences in populations, comparators, exposure definitions, and duration of follow-up, the survival associations do not imply causality or direct antitumor effects of GLP-1 RAs. Clinically, the preponderance of evidence does not support the use of GLP-1 RAs as cancer-directed therapy, and their use in patients with cancer should be guided by established indications such as obesity and type 2 diabetes. Potential explanations include effects on glycemic and cardiovascular risk factors, treatment selection, residual confounding, or combinations thereof. Prospective studies and randomized controlled trials with cancer-specific outcomes and longer follow-up are needed to further evaluate these associations.

## 1. Introduction

Obesity is one of the most prevalent chronic diseases worldwide [1,2]. In 2022, the global prevalence of obesity among adults was estimated at 16.06%, more than double that recorded in 1990, while overweight prevalence reached 28.00% [3]. This burden is expected to increase further, with the global prevalence of obesity projected to reach 38.96% by 2040 if current trends continue [3]. Deaths attributable to high body mass index (BMI) increased from 1.46 million in 1990 to 3.71 million in 2021 [4].

Excess body weight is also an established risk factor for cancer. In 2016, the International Agency for Research on Cancer (IARC) Working Group reviewed more than 1000 epidemiological studies and concluded that sufficient evidence linked excess body fatness to 13 cancer types, including esophageal adenocarcinoma, postmenopausal breast cancer, and cancers of the colon and rectum, kidney, liver, pancreas, thyroid, and corpus uteri. For certain cancer types, the risk increases progressively with BMI; for example, the relative risk of endometrial cancer increases from approximately 1.5 among individuals with overweight to 7.1 among those with class 3 obesity [5]. In 2022, an estimated 547,223 new cancer cases were attributable to obesity worldwide [6].

Several mechanisms link obesity to cancer, many of which arise from adipose tissue dysfunction. In obesity, adiponectin levels decrease, reducing its insulin-sensitizing and tumor-suppressive effects, whereas increased aromatase activity raises estrogen production and may promote the growth of hormone-sensitive tumors [7,8,9]. Adipocyte hypertrophy also promotes chronic low-grade inflammation characterized by increased levels of leptin and proinflammatory cytokines, including interleukin-1β (IL-1β), interleukin-6 (IL-6), and tumor necrosis factor-α (TNF-α). These changes alter macrophage function and reduce the abundance and cytotoxic activity of natural killer (NK) cells and cytotoxic T cells, limiting their ability to eliminate tumor cells [7,9]. Enlarged adipocytes can also supply tumor cells with free fatty acids that serve as energy sources and support tumor growth [7,9]. In parallel, insulin resistance and hyperinsulinemia activate the insulin/insulin-like growth factor-1 (IGF-1) signaling pathway, which may promote tumor progression, particularly in colorectal cancer [8,9]. Obesity-related oxidative stress and impaired deoxyribonucleic acid (DNA) repair further contribute to genomic instability [7,9].

The effects of obesity do not end at cancer diagnosis. Among individuals with an established malignancy, obesity has been associated with poorer overall survival (OS) (hazard ratio [HR] 1.14, 95% confidence interval [CI] 1.09–1.19; *p* < 0.001), poorer cancer-specific survival (HR 1.17, 95% CI 1.12–1.23; *p* < 0.001), and a higher risk of cancer recurrence (HR 1.13, 95% CI 1.07–1.19; *p* < 0.001) [10]. These associations highlight the potential metabolic and prognostic importance of weight management.

Glucagon-like peptide-1 receptor agonists (GLP-1 RAs) have changed obesity management. These therapeutic agents enhance glucose-dependent insulin secretion, inhibit glucagon release, delay gastric emptying, and increase satiety, thereby promoting weight loss [11]. Beyond their effects on glycemic control and body weight, meta-analyses of randomized trials have shown that GLP-1 RAs reduce major adverse cardiovascular events (MACE) and improve kidney outcomes, particularly in patients with type 2 diabetes or elevated cardiovascular risk or chronic kidney disease [12,13].

As the use of GLP-1 RAs has expanded, their potential effects on cancer risk have received increasing attention. Early safety concerns were based on rodent studies reporting thyroid C-cell hyperplasia following long-term liraglutide exposure [14]. In humans, the evidence remains unclear. A meta-analysis of 45 trials reported no significant association between GLP-1 RA use and thyroid cancer [15]. By contrast, a nationwide French case–control analysis of 2562 thyroid cancer cases observed a higher risk of thyroid cancer, including medullary thyroid carcinoma, particularly after one to three years of treatment [16]. However, the study did not adjust for obesity, and detection bias related to potentially increased thyroid monitoring or neck imaging among GLP-1 RA users could not be ruled out [17].

Experimental studies suggest that GLP-1 RAs may affect tumor biology through several mechanisms [18]. Anti-proliferative and anti-inflammatory effects have been described in pancreatic, colorectal, breast, and prostate cancer models, although potential proangiogenic effects have raised concerns [19]. In contrast, semaglutide promoted proliferation in neuroendocrine neoplasm cell lines expressing glucagon-like peptide-1 receptors and increased tumor growth in a mouse xenograft model [20]. These effects appear to vary according to receptor expression, tumor type, drug, dose, and the characteristics of the cells involved [20,21].

Previous clinical research has mainly examined the association between GLP-1 RA use and cancer incidence, whereas clinical outcomes after cancer diagnosis have received considerably less attention [18,19,22]. Meanwhile, as GLP-1 RA use expands, an increasing number of patients newly diagnosed with cancer are already receiving these agents for obesity or type 2 diabetes [23]. These patients represent a clinically relevant population in which obesity itself is associated with poorer survival and a higher risk of recurrence [10]. Prospective evidence to guide these treatment decisions remains limited [18].

Within this review, GLP-1 RA was the exposure of interest. Other glucose-lowering agents were included solely as comparator treatments in the primary studies, and these medications were not considered mechanistically equivalent exposures. The rationale for including active-comparator studies was to provide clinically relevant comparisons between alternative metabolic therapies and to supplement comparisons with GLP-1 RA nonusers.

The primary objective of this systematic review was to determine the association between selective GLP-1 receptor agonist use and overall survival or all-cause mortality in adult patients with an established malignancy in comparison with GLP-1 receptor agonist nonuse or active non-GLP-1 comparator therapy. Secondary objectives were to evaluate associations with cancer-specific mortality, cancer recurrence, disease progression, and other cancer-related outcomes.

## 2. Methods

### 2.1. Protocol and Reporting Guidelines

This systematic review was conducted and reported in accordance with the Preferred Reporting Items for Systematic Reviews and Meta-Analyses (PRISMA) 2020 statement [24]. The review was not registered, and no a priori protocol was prepared. The review question and eligibility criteria were defined before study screening and data extraction.

### 2.2. Eligibility Criteria

Eligible studies included adult (≥18 years of age) patients with a confirmed cancer diagnosis receiving GLP-1 RA treatment and measured at least one clinically relevant oncologic or survival outcome specified in the primary or secondary objectives. Studies of selective GLP-1 RAs were eligible even when analyzed together with dual GLP-1/glucose-dependent insulinotropic polypeptide (GIP) receptor agonists, as they included patients receiving the exposure of interest and assessed oncologic or survival outcomes relevant to the review objectives.

Eligible comparator groups included patients with no or minimal GLP-1 RA exposure and patients receiving an active comparator. Eligible outcomes included overall survival (OS), all-cause mortality, cancer-related or cancer-specific mortality, disease-free survival (DFS), progression-free survival (PFS), recurrence-free survival (RFS), cancer recurrence, locoregional recurrence (LRR), metastatic disease, disease progression, response-to-therapy status, and no evidence of disease (NED) status. Studies were included regardless of follow-up duration. Outcomes outside the oncologic and survival categories considered in this review did not determine study eligibility and were not included in the formal synthesis.

For the main qualitative synthesis, only original comparative studies conducted in humans and published in English between 1 January 2016, and 8 August 2026, were eligible. Reviews and other non-original publications, non-comparative clinical reports, conference abstracts without a corresponding full-text publication, preclinical studies, and studies that did not report an eligible outcome were excluded. Studies evaluating cancer incidence or the risk of developing cancer in populations without an established cancer diagnosis were also excluded.

### 2.3. Information Sources

A systematic literature search was conducted in three electronic databases: PubMed, Scopus, and Web of Science. All three databases were searched on 8 August 2026.

A supplementary search of ClinicalTrials.gov and the World Health Organization International Clinical Trials Registry Platform (WHO ICTRP) was conducted on 9 October 2026, during manuscript revision. This search aimed to identify ongoing trials and potentially unpublished clinical evidence evaluating GLP-1–based therapies in patients with an established malignancy.

### 2.4. Search Strategy

The bibliographic search strategy involved the use of Medical Subject Headings (MeSH) terms, where available, and free-text keywords related to GLP-1 RAs, cancer, mortality, survival, recurrence and progression. Individual drug names and common terms for the GLP-1 RA class were also included. Terms within each group were combined using the Boolean operator OR, and the three groups were combined using AND. The language and publication-date limits were applied in each database. The registry searches combined cancer-related terms with GLP-1 receptor agonist class terms and individual drug names, including tirzepatide. No filters were applied to restrict the registry search results. The complete database- and registry-specific search strategies are provided in Appendix A.

### 2.5. Study Selection Process

**Figure 1:**
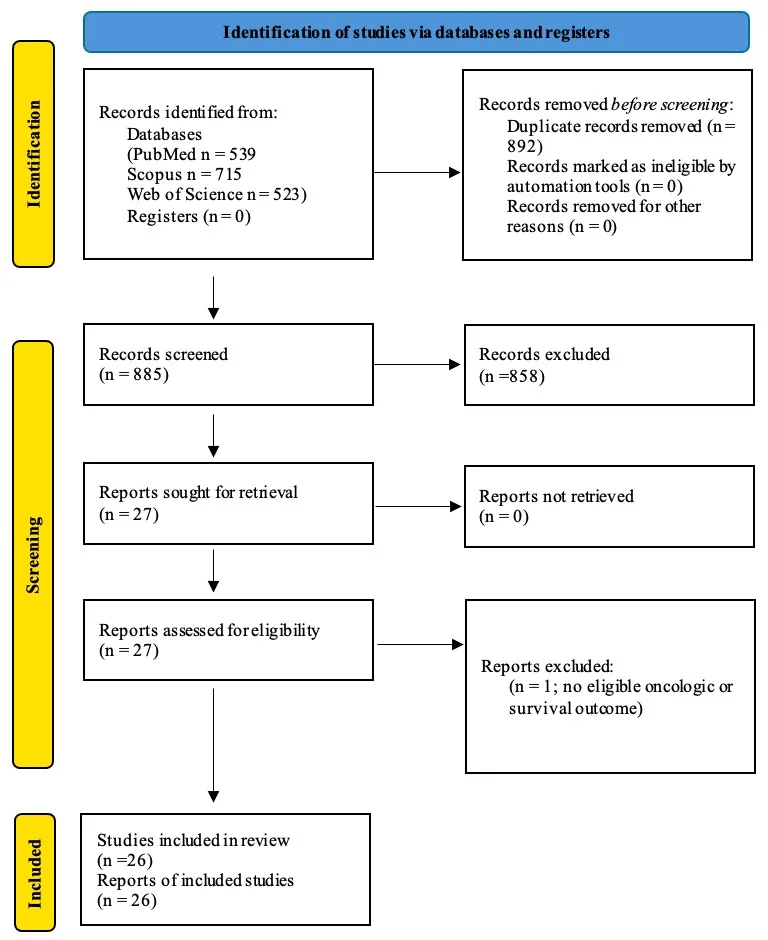
Preferred Reporting Items for Systematic Reviews and Meta-Analyses (PRISMA) 2020 flow diagram of study selection.

Records retrieved from the bibliographic databases were exported to Zotero reference management software (version 9.0.6), and duplicate records were identified and removed. Two reviewers independently screened the titles and abstracts according to the eligibility criteria and subsequently assessed the full texts of potentially eligible publications. Disagreements arising during study selection, data extraction, or risk-of-bias assessment were resolved through discussion and consensus. The selection of published studies retrieved from the bibliographic databases is summarized in the PRISMA flow diagram (Figure 1).

### 2.6. Data Extraction

Two reviewers independently performed data extraction from each included study using a standardized form developed for this review. Extracted information included the first author and publication year, country and data source, study period and design, population characteristics, cancer type, sample size, exposure and comparator groups, primary and secondary outcomes, effect estimates including hazard ratios (HRs), odds ratios (ORs), and risk ratios (RRs), with 95% CIs, *p*-values, follow-up duration, main findings, and study limitations.

Data were collected for all eligible oncologic and survival outcomes. When a study reported multiple cohorts, comparator groups, or analyses, the relevant results were extracted separately. Outcomes were classified as primary, secondary, or exploratory where specified in the original studies, and sensitivity analyses were identified as such. Effect estimates were recorded as reported by the study authors; data items not provided in the source publication were recorded as not reported (NR).

### 2.7. Risk of Bias Assessment Process

Methodological quality and risk of bias were independently assessed by two reviewers using the Newcastle–Ottawa Scale (NOS) for cohort studies [25]. The NOS assessed three domains: selection of the study groups, comparability of the cohorts, and outcome assessment, with a maximum score of 9 points. Studies scoring 7–9 points were classified as high quality, those scoring 5–6 points as moderate quality and those scoring fewer than 5 points as low quality [25].

### 2.8. Data Synthesis

For the qualitative synthesis, studies were grouped according to cancer type and the outcomes evaluated. Findings were summarized descriptively using the reported effect estimates and 95% CIs. Findings were interpreted according to comparator type, exposure definition, and the hierarchy and context of the original analyses rather than as independent confirmatory results.

Studies with mixed incretin exposure were identified as such in the narrative synthesis and summary tables. Drug-specific estimates were presented individually where available. Pooled estimates combining selective GLP-1 RAs and dual GLP-1/GIP receptor agonists were not used to support class-specific conclusions regarding selective GLP-1 RAs.

HRs, RRs and ORs were interpreted as distinct measures of association. HRs compare the instantaneous rates of an event during follow-up among patients who have not yet experienced it. RRs compare the probability of an event over a specified period, whereas ORs compare event odds, defined as the probability of the event divided by the probability of its absence. Therefore, identical numerical values of these measures do not indicate equivalent differences in risk. In particular, HRs and ORs were not interpreted as percentage reductions in cumulative risk. The direction of each association was interpreted according to the outcome and reference group reported in the original study, and the numerical magnitudes of different effect measures were not treated as directly equivalent.

A meta-analysis was not performed because the studies differed substantially in cancer type, study populations, exposure and comparator definitions, outcomes, follow-up duration and analytical methods.

The supplementary registry search was conducted to provide information on ongoing research and potentially unpublished evidence and did not contribute outcome estimates to the main qualitative synthesis.

## 3. Results

### 3.1. Study Selection

A total of 1777 records were identified through database searches, including 539 records from PubMed, 715 from Scopus, and 523 from Web of Science. After removing 892 duplicate records, 885 records underwent title and abstract screening, of which 858 records were excluded. The full texts of the remaining 27 reports were assessed for eligibility. One report was excluded because it did not report an eligible oncologic or survival outcome. Ultimately, 26 studies met the eligibility criteria and were included in the qualitative synthesis. The study selection process is presented in Figure 1.

### 3.2. Study Characteristics

The 26 included studies were published between 2025 and 2026 and were all retrospective cohort studies. Seventeen studies used the TriNetX federated real-world network [26,27,28,29,30,31,32,33,34,35,36,37,38,39,40,41,42]. Other data sources included institutional or health-system records [43,44,45,46,47,48], Medicare claims data [49], and Chinese multicenter or national healthcare databases [50,51].

Most studies were reported as full-length original articles. One was published as a Brief Communication [47], and two were published as Research Letters [42,49]; all three reported original comparative data.

Study populations covered a broad range of malignancies. Site-specific cohorts included bladder cancer [26], colorectal or colon cancer [27,45], ovarian, fallopian tube, or primary peritoneal carcinoma [28], differentiated thyroid cancer [43], non-small cell or other lung cancer [44,51], breast cancer [31,37,38,46,47], neuroendocrine neoplasms [32], hepatocellular carcinoma [50], and renal cell carcinoma [41]. Eleven studies included patients with different solid or hematologic malignancies [29,30,33,34,35,36,39,40,42,48,49].

Cohort sizes varied widely, with GLP-1 RA-exposed groups ranging from 10 to 19,803 patients [34,44]. Propensity score matching was used in 23 studies, while the other three used direct covariate matching [43], inverse probability of treatment weighting [50], or multivariable adjustment without matching or weighting [51].

Comparator strategies varied across studies, and several studies included more than one comparison. Nineteen studies used comparator groups comprising patients classified as GLP-1 RA nonusers [26,28,29,31,32,34,35,36,39,40,41,42,43,44,45,46,47,48,51]. In one of these studies, patients with less than 28 days of GLP-1 RA use were included in the nonuser group [51]. Three studies used broader comparator groups receiving glucose-lowering regimens without a GLP-1 RA [27,33,37]. Specific active-comparator analyses included metformin [30,31,39], insulin [30,31,42], sodium-glucose cotransporter 2 (SGLT2) inhibitors [31,33,42,49], dipeptidyl peptidase-4 (DPP-4) inhibitors [33,42,49,50], sulfonylureas [39,42], and thiazolidinediones [42]. Bariatric surgery was used as a comparator in one study [38]. Thirteen studies were identified as mixed incretin-exposure cohorts because the authors of the original studies included selective GLP-1 RAs and tirzepatide within the same exposure group [28,30,31,32,33,35,37,38,39,42,43,45,46].

Detailed study characteristics are presented in Table 1.

### 3.3. Risk of Bias Assessment

The methodological quality of all included studies was assessed using the Newcastle–Ottawa Scale (NOS) [25], with scores ranging from 6 to 9 points. One study achieved the maximum score of 9 points, 20 studies scored 8 points, 4 studies scored 7 points, and one study scored 6 points. Twenty-five studies were classified as high quality, while one was classified as moderate quality. Points were most often deducted in the outcome domain because follow-up completeness was not adequately reported in 22 studies. Seven studies also lost one point for limited cohort representativeness, while two received only one comparability point because of incomplete control of confounding or residual imbalance after matching. These ratings reflect the domains captured by the NOS and should not be interpreted as evidence that all relevant sources of bias were absent. The detailed quality assessment is presented in Table 2.

### 3.4. Overall Survival and Mortality Outcomes

Twenty-five studies evaluated the association between GLP-1 RA use, including mixed incretin exposure in some cohorts, and OS and/or all-cause mortality in patients with cancer. One of these studies also reported cancer-related mortality [48]. Most studies reported improved survival or lower mortality among patients in the exposed groups, although the strength and statistical significance of the associations varied according to the population, comparator, and analytical model.

**Table 1:** Main characteristics of the included studies.

Author, Year	Country/Database	Study Period	Population	Cancer Type	Sample Size (N)	Exposure Definition and Timing	Comparator
Kohler et al., 2026 [26]	Global/TriNetX Global Collaborative Network	2013–2024	Adults (≥18 years) with evidence of oncologic treatment (surgery, chemotherapy, and/or radiotherapy)	Bladder cancer	After PSM: *N* = 10,588 (5294 GLP-1 RA users; 5294 nonusers)	GLP-1 RA exposure within a prespecified peri-diagnostic window linked to oncologic treatment (surgery, chemotherapy and/or radiotherapy). Agents listed in the supplementary definition: semaglutide, dulaglutide, liraglutide, lixisenatide and exenatide	No GLP-1 RA use within the corresponding temporal window
Dosda et al., 2026 [27]	International/TriNetX Research Network	January 2010–January 2025	Adults aged 18–80 years with T2D	Newly diagnosed colorectal cancer	After PSM: *N* = 1502 (751 GLP-1 RA users; 751 controls)	GLP-1 RA exposure within 6 months of CRC diagnosis, with ≥1 prescription between 1 week and 6 months after diagnosis; included pre-diagnosis use continued after diagnosis. Individual agents were not specified	Other non-insulin glucose-lowering therapies
Wang M et al., 2025 [28]	Global/TriNetX Research Network	January 2014–December 2023	Women aged 18–75 years with no history of other malignancies	Ovarian, fallopian tube and primary peritoneal carcinoma	After PSM and 3-month exclusion: *N* = 1966 (1007 GLP-1 RA users; 959 nonusers)	GLP-1 RA or tirzepatide use for ≥1 month within 6 months before or after cancer diagnosis (mixed incretin-exposure cohort)	No GLP-1 RA use
Patrizio et al., 2026 [43]	USA/Memorial Sloan Kettering Cancer Center	January 2005–December 2024	Adults (≥18 years) with DM and/or obesity	Differentiated thyroid cancer	Overall matched cohort: *N* = 1072 (536 GLP-1 RA users; 536 nonusers); main survival subcohort: *N* = 840 (420 users; 420 nonusers)	GLP-1 RA or tirzepatide use for diabetes or obesity (mixed incretin-exposure cohort); prescription-defined treatment intervals, modeled as time-dependent exposure	No exposure to a GLP-1 RA or dual GLP-1/GIP RA
Chan et al., 2026 [29]	USA/TriNetX US Collaborative Network	1 January 2018–31 August 2025	Adults (≥18 years) with cancer and T2D initiating ICI therapy	Mixed cancers: NSCLC, breast, urothelial, melanoma and other cancers	After PSM: *N* = 5806 (2903 GLP-1 RA users; 2903 nonusers)	Concurrent GLP-1 RA use at ICI initiation; new-user design with a 3-month medication washout	ICI initiation without GLP-1 RA use
Mahadevan et al. 2026 [30]	Global/TriNetX Global Collaborative Network	1 January 2010 onward	Patients with T2D and active cancer receiving systemic anticancer therapy	Selected solid and hematologic malignancies	After 1:1 PSM: overall cohort, *N* = 7102 (3551 GLP-1 RA; 3551 metformin); new-start cohort, *N* = 3240 (1620 GLP-1 RA; 1620 insulin)	GLP-1 RA or tirzepatide use within ±3 months of systemic anticancer therapy initiation (mixed incretin-exposure cohort): overall cohort, without concurrent metformin; new-start cohort, first recorded use in prior metformin users	Metformin monotherapy; new-start insulin in metformin users
Pachimatla et al. 2025 [44]	USA/Roswell Park Comprehensive Cancer Center medical records	Postsurgical cohort: 2015–2024; advanced-disease cohort: 2015–2023	Overweight or obese patients with histologically confirmed NSCLC undergoing surgical resection or receiving ICI therapy for advanced disease	Non-small cell lung cancer	After PSM: postsurgical cohort, *N* = 629 (69 GLP-1 RA users; 560 nonusers); advanced-disease cohort, *N* = 89 (10 GLP-1 RA users; 79 nonusers)	GLP-1 RA use for >6 months after resection (postsurgical cohort) or during ICI therapy (advanced-disease cohort); individual agents were not specified	No GLP-1 RA use
Tatum et al. 2026 [31]	USA/TriNetX US Collaborative Network	1 April 2006–1 April 2023	Adult women (≥18 years) with stage I–III breast cancer, including an obesity cohort without T2D and a T2D cohort	Breast cancer	After 1:1 PSM: obesity cohort, *N* = 3136–3220; T2D GLP-1 RA vs. insulin/metformin cohort, *N* = 4624–4646; T2D GLP-1 RA vs. SGLT2i cohort, *N* = 8000–8104 (sample size varied by outcome)	GLP-1 RA or tirzepatide use, defined as ≥2 prescription records within 6 months before or at any time after breast cancer diagnosis (mixed incretin-exposure cohort)	GLP-1 RA nonuse or other weight-loss medication in the obesity cohort; insulin or metformin and SGLT2i in the T2D cohort
Fawzy et al. 2025 [32]	USA/TriNetX US Research Network	NR	Adults (≥18 years) with cancer and concomitant diabetes mellitus or obesity	Neuroendocrine neoplasms	After PSM: *N* = 6086 (3043 GLP-1 RA users; 3043 nonusers)	GLP-1 RA or tirzepatide use, defined as ≥1 prescription within 1 year after NEN diagnosis (mixed incretin-exposure cohort)	No GLP-1 RA therapy
Cuomo 2025 [45]	USA/University of California Health Data Warehouse (UCHDW)	Colon cancer diagnosis before 1 January 2019	Patients with primary colon cancer	Colon cancer	After PSM: *N* = 1708 (103 GLP-1 RA users; 1605 nonusers)	GLP-1 RA or tirzepatide use, defined as ≥1 recorded drug exposure within 5 years after colon cancer diagnosis (mixed incretin-exposure cohort)	No GLP-1 RA exposure within 5 years after diagnosis
Sukumar et al. 2026 [46]	USA/MD Anderson Cancer Center Breast Medical Oncology Management System and institutional pharmacy database	January 2005–January 2024	Adults aged ≥ 18 years with stage I–III invasive breast cancer	Breast cancer	After PSM: *N* = 2430 (810 GLP-1 RA users; 1620 nonusers)	GLP-1 RA or tirzepatide use for ≥90 consecutive days, predominantly initiated after breast cancer diagnosis (mixed incretin-exposure cohort)	No prior GLP-1 RA use
Chiang et al. 2025 [33]	NR/TriNetX Analytics Network	April 2013–May 2023	Adults with cancer and T2D receiving ICI therapy	Multiple solid and hematologic malignancies	After PSM: *N* = 938 (469 GLP-1 RA users; 469 nonusers)	GLP-1 RA or tirzepatide use, defined as ≥1 prescription within 12 months before ICI initiation and ≥1 prescription afterward (mixed incretin-exposure cohort)	Non-GLP-1 RA antihyperglycemic therapy; sensitivity analyses: SGLT2i and DPP-4i
Radwan et al. 2025 [49]	USA/Medicare claims data	2013–2020	Adults aged ≥ 66 years with T2D and cancer who initiated a study drug and survived ≥ 1 year after cancer diagnosis	Multiple solid malignancies	After PSM: GLP-1 RA vs. SGLT2i, *N* = 5106 (2553/group); GLP-1 RA vs. DPP-4i, *N* = 5128 (2564/group)	GLP-1 RA initiation in patients with established cancer; index date defined as the first prescription (intention-to-treat analysis)	SGLT2i initiation or DPP-4i initiation in separate active-comparator cohorts
Nelson et al. 2026 [47]	USA/City of Hope Cancer Registry and institutional medical records	2009–2025	Women with T2D and stage I–IV invasive breast cancer	Breast cancer	After PSM: *N* = 452 (226 GLP-1 RA users; 226 nonusers)	Documented GLP-1 RA use after breast cancer diagnosis; individual agents were not specified	No post-diagnosis GLP-1 RA use
Javaid et al. 2026 [34]	Global, primarily USA/TriNetX database	January 2010–December 2023	Adults (aged ≥ 18 years) with cancer receiving cardiotoxic antineoplastic therapy without pre-existing HF, cardiomyopathy or LVEF ≤ 50%	Multiple solid and hematologic malignancies	After PSM: *N* = 39,606 (19,803 GLP-1 RA users; 19,803 nonusers)	GLP-1 RA initiation during ongoing cardiotoxic antineoplastic therapy; individual agents were not specified	No GLP-1 RA use during cardiotoxic antineoplastic therapy
Scalia et al. 2025 [35]	International/TriNetX Research Network	January 2012–December 2022	Adults (aged ≥ 18 years) with anthracycline-induced CTRCD	Cancer types NR	After PSM: *N* = 402 (201 GLP-1 RA users; 201 nonusers)	Active GLP-1 RA or tirzepatide use, initiated before or at diagnosis of anthracycline-related cardiomyopathy (mixed incretin-exposure cohort)	No GLP-1 RA use in patients with anthracycline-induced CTRCD
Vignarajah et al. 2025 [36]	Global/TriNetX Research Network	January 2012–January 2023	Adults (aged ≥ 18 years) with cancer who developed CTRCD after cardiotoxic antineoplastic therapy	Multiple solid and hematologic malignancies	After PSM: *N* = 1674 (837 GLP-1 RA users; 837 nonusers)	GLP-1 RA initiation after CTRCD diagnosis (new users); individual agents were not specified	No GLP-1 RA use in patients with CTRCD
Cornelio et al. 2026 [37]	Global/TriNetX Global Collaborative Network	16 December 2014–16 December 2024	Women with T2D who were aged ≥ 40 years at cancer diagnosis	Breast cancer	After PSM: *N* = 39,216 (19,608/group); all-cause mortality analysis: *N* = 39,061 (19,530 GLP-1 RA; 19,531 non-GLP-1 RA).	GLP-1 RA or tirzepatide use at or within 5 years after breast cancer diagnosis (mixed incretin-exposure cohort)	Non-GLP-1 RA diabetes medication use during the same period
Den et al. 2026 [38]	USA/TriNetX U.S. Collaborative Network	NR	Women aged ≥ 50 years with obesity	Stage 0 (DCIS)–III breast cancer	After PSM: Study 1, *N* = 6876 (3438/group); Study 2, *N* = 2258 (1129/group)	Study 1: GLP-1 RA or tirzepatide use ≥ 6 months after diagnosis (mixed incretin-exposure cohort) Study 2: GLP-1 RA or tirzepatide use combined with bariatric surgery ≥ 6 months after diagnosis (mixed incretin-exposure cohort)	Bariatric surgery ≥ 6 months after diagnosis without prior GLP-1 RA use
Xiang et al. 2026 [50]	China/nationwide multicentre EMR collaboration comprising 36 participating centers	1 January 2014–31 December 2023	Adults with T2D who underwent curative-intent R0 liver resection	Hepatocellular carcinoma	Final analytical cohort: *N* = 1249 (526 GLP-1 RA initiators; 723 DPP-4i initiators)	GLP-1 RA use initiated within 90 days after R0 resection for HCC (new users)	DPP-4i initiation within 90 days after resection
Chen ST et al. 2026 [48]	USA/Mayo Clinic Electronic Medical Record	2013–2023	Adults aged ≥ 18 years with cancer and T2D receiving anthracycline therapy	Mixed malignancies, including lymphoma, breast cancer, sarcoma and leukemia	After PSM: *N* = 280 (56 GLP-1 RA users; 224 nonusers) Stage-matched cohort: *N* = 138 (46 GLP-1 RA users; 92 nonusers)	Concurrent GLP-1 RA use, initiated before or at anthracycline treatment initiation: exenatide, lixisenatide, semaglutide, dulaglutide and liraglutide	No concurrent GLP-1 RA use
Chen Z et al. 2026 [51]	China/National Healthcare Big Data (East) Center	January 2020–July 2023	Patients with newly diagnosed lung cancer and T2D	Lung cancer	GLP-1 RA analysis: *N* = 5529 (502 users; 5027 nonusers [<28 days of use])	GLP-1 RA use for ≥28 days, categorized by treatment duration (<106, 106–292, 293–559 or ≥560 days); initiation timing relative to cancer diagnosis was not specified	GLP-1 RA use for <28 days or no GLP-1 RA use
Lin et al. 2026 [39]	USA/TriNetX US Collaborative Network	1 January 2017–31 October 2025	Adults (≥18 years) with cancer and overweight/obesity without baseline diabetes who initiated ICI therapy; prediabetes was permitted	Mixed solid malignancies: NSCLC, breast and urothelial cancers, melanoma, and other solid tumors	Primary cohort after PSM: *N* = 1974 (987/group); active-comparator OS analysis: 464 vs. 492 patients at risk at day 0; matched cohort size NR	GLP-1 RA or tirzepatide use, defined by prescription or administration within a 30-day peri-initiation window around ICI initiation, with a 90-day GLP-1 RA washout (mixed incretin-exposure cohort)	Primary cohort: ICI without GLP-1 RA; active-comparator cohort: ICI plus metformin or sulfonylurea
Wu et al., 2026 [40]	Global/TriNetX worldwide federated network	1 January 2017–31 March 2025	Adults (≥18 years) with cancer receiving potentially cardiotoxic antineoplastic therapy	Mixed malignancies: breast, gastrointestinal, respiratory/intrathoracic, urinary, reproductive, hematologic and mesothelial/soft-tissue cancers; metastatic disease of unknown primary	After PSM: *N* = 16,772 (8386 GLP-1 RA users; 8386 nonusers)	GLP-1 RA use initiated within 1 year before or after potentially cardiotoxic anticancer treatment	No GLP-1 RA use during potentially cardiotoxic cancer therapy
Kwon et al. 2026 [41]	Global/TriNetX database	January 2005–November 2025	Adults aged 18–90 years with T2D prescribed antidiabetic medication before partial nephrectomy	Presumed localized renal cell carcinoma	After PSM: *N* = 2420 (1210 GLP-1 RA users; 1210 nonusers)	GLP-1 RA use defined by prescription records in the perioperative setting of partial nephrectomy; precise exposure timing and duration were not reported	No GLP-1 RA use
Chi et al. 2026 [42]	Global/TriNetX Global Network	1 January 2018–1 January 2024	Adults aged ≥ 18 years with cancer, brain metastases and T2D	Mixed primary cancers with brain metastases, mainly lung cancer, breast cancer and melanoma	After PSM: *N* = 1700 (850/group)	GLP-1 RA or tirzepatide use, defined by prescription within 6 months before the first qualifying diagnosis of T2D and brain metastases (mixed incretin-exposure cohort)	No GLP-1 RA use during the same period; additional active-comparator analyses included DPP-4 inhibitors, SGLT2 inhibitors, sulfonylureas, thiazolidinediones, and insulin.

CRC, colorectal cancer; CTRCD, cancer therapy-related cardiac dysfunction; DCIS, ductal carcinoma in situ; DM, diabetes mellitus; DPP-4i, dipeptidyl peptidase-4 inhibitor; EMR, electronic medical record; GIP, glucose-dependent insulinotropic polypeptide; GLP-1 RA, glucagon-like peptide-1 receptor agonist; HCC, hepatocellular carcinoma; HF, heart failure; ICI, immune checkpoint inhibitor; LVEF, left ventricular ejection fraction; *N*, sample size; NEN, neuroendocrine neoplasm; NR, not reported; NSCLC, non-small cell lung cancer; OS, overall survival; PSM, propensity score matching; R0, resection with microscopically negative margins; RA, receptor agonist; SGLT2i, sodium-glucose cotransporter 2 inhibitor; T2D, type 2 diabetes; UCHDW, University of California Health Data Warehouse; US/USA, United States of America; vs., versus.

**Table 2:** Newcastle–Ottawa Scale assessment of the included studies.

Author/Year	Study Design	Selection (0–4)	Comparability (0–2)	Outcome (0–3)	Total NOS Score (0–9)	Quality Category (High/Moderate/Low)
Kohler et al., 2026 [26]	Retrospective	4	2	2	8	High
Dosda et al., 2026 [27]	Retrospective	4	2	2	8	High
Wang et al., 2025 [28]	Retrospective	4	2	2	8	High
Patrizio et al. 2026 [43]	Retrospective	3	2	3	8	High
Chan et al., 2026 [29]	Retrospective structured as a TTE	4	2	2	8	High
Mahadevan et al., 2026 [30]	Retrospective	4	2	2	8	High
Pachimatla et al., 2025 [44]	Retrospective	3	1	2	6	Moderate
Tatum et al., 2026 [31]	Retrospective	4	2	2	8	High
Fawzy et al., 2025 [32]	Retrospective	4	2	2	8	High
Cuomo, 2025 [45]	Retrospective	3	2	3	8	High
Sukumar et al., 2026 [46]	Retrospective	3	2	2	7	High
Chiang et al., 2025 [33]	Retrospective	4	2	2	8	High
Radwan et al., 2025 [49]	Retrospective	4	2	3	9	High
Nelson et al., 2026 [47]	Retrospective; with a 2-year landmark analysis	3	2	3	8	High
Javaid et al., 2026 [34]	Retrospective	4	2	2	8	High
Scalia et al., 2025 [35]	Retrospective	4	2	2	8	High
Vignarajah et al., 2025 [36]	Retrospective	4	2	2	8	High
Cornelio et al., 2026 [37]	Retrospective	4	2	2	8	High
Den et al., 2026 [38]	Retrospective cohort	4	2	2	8	High
Xiang et al., 2026 [50]	Retrospective; structured as a TTE	4	2	2	8	High
Chen ST et al., 2026 [48]	Retrospective	3	2	2	7	High
Chen Z et al., 2026 [51]	Retrospective	3	2	2	7	High
Lin et al., 2026 [39]	Retrospective; structured as a TTE	4	2	2	8	High
Wu et al., 2026 [40]	Retrospective	4	2	2	8	High
Kwon et al., 2026 [41]	Retrospective	4	2	2	8	High
Chi et al., 2026 [42]	Retrospective	4	1	2	7	High

NOS, Newcastle–Ottawa Scale; TTE, target trial emulation.

Among studies of specific cancer types, Kohler et al. reported improved OS among GLP-1 RA users with bladder cancer, with fewer all-cause deaths than among nonusers. The association persisted in both landmark sensitivity analyses. The proportional hazards assumption was not met in the main analysis; the authors therefore interpreted the HR as a time-averaged estimate [26]. Lower all-cause mortality was also observed among GLP-1 RA users with colorectal cancer compared with patients receiving other non-insulin glucose-lowering therapies, as reported by Dosda et al. [27]. Wang et al. reported lower all-cause mortality among women with ovarian, fallopian tube, or primary peritoneal carcinoma receiving GLP-1 RAs or tirzepatide compared with nonusers in a mixed incretin-exposure cohort. The association persisted in adjusted, sensitivity, and landmark analyses. Drug-specific comparisons with nonusers were statistically significant for semaglutide and dulaglutide, but not for liraglutide or tirzepatide [28]. Similarly, in a mixed incretin-exposure cohort of patients with neuroendocrine neoplasms and diabetes or obesity, Fawzy et al. reported lower all-cause mortality among GLP-1 RA or tirzepatide users compared with nonusers. Drug-specific comparisons with nonusers were statistically significant for semaglutide, dulaglutide, and tirzepatide, but not for liraglutide or exenatide [32]. In colon cancer, Cuomo found significantly lower odds of five-year mortality among GLP-1 RA or tirzepatide users compared with nonusers in a mixed incretin-exposure cohort, but the association with time to death was not significant in the overall Cox models. The association with five-year mortality remained significant across carcinoembryonic antigen (CEA)—based sensitivity models. In BMI-stratified analyses, significant associations for both outcomes were observed only in the highest BMI category [45]. Pachimatla et al. observed OS estimates favoring GLP-1 RA use over nonuse in both the postsurgical and advanced non-small cell lung cancer (NSCLC) cohorts, although the 95% CIs remained compatible with no difference between groups. The clinical analyses were exploratory, and the reported *p*-values for survival outcomes were one-sided [44]. Similarly, in patients with lung cancer, Chen Z et al. reported that none of the GLP-1 RA treatment-duration categories was significantly associated with OS compared with the untreated group in fully adjusted analyses. An additional spline analysis of mortality, designated as a sensitivity analysis, reported an overall association with treatment duration; its covariate-adjustment model was not explicitly specified [51].

Five breast cancer studies reported survival outcomes. Tatum et al. found lower all-cause mortality with GLP-1 RA or tirzepatide use in mixed incretin-exposure cohorts compared with nonuse and insulin or metformin; however, the comparison with SGLT2 inhibitors was nonsignificant in the unadjusted matched analysis and became significant only after further adjustment. In the landmark analyses, mortality differences versus nonuse and SGLT2 inhibitors were nonsignificant, whereas the association versus insulin or metformin remained significant [31]. Similarly, Sukumar et al. reported significantly improved OS with GLP-1 RA or tirzepatide use in a mixed incretin-exposure cohort compared with nonuse. The OS association remained statistically significant across all landmark analyses [46]. Nelson et al. also found lower all-cause mortality with GLP-1 RA use compared with nonuse in a prespecified landmark analysis. The association remained statistically significant in the sensitivity analysis excluding patients with stage IV disease [47]. Cornelio et al. observed lower five-year all-cause mortality with GLP-1 RA or tirzepatide use in a mixed incretin-exposure cohort compared with other glucose-lowering therapies. All-cause mortality was a secondary outcome, whereas MACE was the primary outcome [37]. Den et al. found lower all-cause mortality both for GLP-1 RA or tirzepatide use compared with bariatric surgery alone and for GLP-1 RA or tirzepatide use combined with bariatric surgery compared with bariatric surgery alone. Both analyses involved mixed incretin-exposure cohorts [38].

In populations receiving systemic anticancer treatment, Chan et al. reported lower all-cause mortality among patients with type 2 diabetes receiving immune checkpoint inhibitors (ICIs) with concurrent GLP-1 RA use at the start of immunotherapy than among nonusers. The mortality association persisted in the landmark analysis, Global Network replication, and additional matching for ICI class. Analyses restricted to semaglutide or to the combined liraglutide/dulaglutide group also showed lower mortality compared with nonuse [29]. Mahadevan et al. also studied patients with type 2 diabetes and active cancer receiving chemotherapy, ICIs or targeted therapies. They observed lower mortality with GLP-1 RA use in two separate analyses of mixed incretin-exposure cohorts: compared with metformin monotherapy in the overall cohort and with insulin initiation among metformin users in the new-start cohort. Both main analyses used a landmark design. However, in the overall cohort, the survival associations were not statistically significant in analyses stratified by glycated hemoglobin (HbA1c) or obesity status [30]. In patients with type 2 diabetes receiving ICIs, Chiang et al. found lower all-cause mortality, evaluated as a safety outcome, among GLP-1 RA or tirzepatide users in a mixed incretin-exposure cohort than among those receiving other glucose-lowering medications. In sensitivity analyses using specific active comparators, the association remained significant with DPP-4 inhibitors as the comparator, but not with SGLT2 inhibitors [33]. Among patients with cancer and overweight or obesity but without diabetes, Lin et al. reported lower all-cause mortality among those who received a GLP-1 RA or tirzepatide in a mixed incretin-exposure cohort around the initiation of immune checkpoint inhibitor (ICI) therapy than in the unexposed group. Mortality was a contextual secondary endpoint, whereas the wasting-related composite was the primary outcome. The authors interpreted the mortality association as exploratory and considered channeling bias its most likely explanation. A separate active-comparator analysis also showed higher OS with ICIs plus GLP-1 RAs or tirzepatide than with ICIs plus metformin or a sulfonylurea, but involved a cohort distinct from the diabetes-free primary cohort and was considered supportive only [39].

Among studies focused on patients receiving potentially cardiotoxic cancer treatment, Javaid et al., Vignarajah et al., and Wu et al. reported lower all-cause mortality among GLP-1 RA users than among nonusers [34,36,40]. In Javaid et al., all-cause mortality was a secondary outcome, whereas CTRCD was the primary outcome. Lower mortality was also observed in the metastatic cancer subgroup [34]. In Wu et al., all-cause mortality was a secondary outcome, whereas cancer therapy-related cardiac dysfunction (CTRCD) was the primary outcome; no adjustment was made for multiple comparisons [40]. In contrast, Scalia et al. found no statistically significant difference in all-cause mortality between GLP-1 RA or tirzepatide users and nonusers in a mixed incretin-exposure cohort of patients with anthracycline-induced cardiomyopathy [35]. In a separate cohort of patients with type 2 diabetes treated with anthracyclines, Chen ST et al. observed significantly lower all-cause and cancer-related mortality among GLP-1 RA users than among matched nonusers in both the overall and stage-matched cohorts. All-cause mortality was the primary outcome, whereas cancer-related mortality was secondary. However, in the subgroup treated with a GLP-1 RA for at least three months before anthracycline therapy, the association with lower all-cause mortality was no longer statistically significant after multivariable adjustment, and cancer-related mortality did not differ significantly between groups [48].

In a cohort of adults aged ≥ 66 years with cancer and type 2 diabetes who had survived at least one year after cancer diagnosis, Radwan et al. found lower all-cause mortality with GLP-1 RA use compared with DPP-4 inhibitors, but no significant difference compared with SGLT2 inhibitors [49].

Following curative-intent resection for hepatocellular carcinoma, Xiang et al. reported longer OS among patients with type 2 diabetes who initiated a GLP-1 RA than among those who initiated DPP-4 inhibitors in the weighted intention-to-treat analysis. OS was a secondary outcome. In the prespecified per-protocol analysis, RFS and OS findings were directionally consistent, but the proportional hazards assumption was not supported for either outcome; interpretation therefore emphasized time-specific estimates and restricted mean survival time (RMST) differences [50]. Kwon et al. similarly observed that GLP-1 RA users had improved OS after partial nephrectomy for renal cell carcinoma in patients with type 2 diabetes compared with nonusers. Postoperative complications were the primary outcomes, while OS was also evaluated without an explicit primary or secondary designation [41]. Chi et al. found lower all-cause mortality, the primary outcome, among GLP-1 RA or tirzepatide users in a mixed incretin-exposure cohort with brain metastases and type 2 diabetes than among matched nonusers. Findings were consistent across the reported cancer-site subgroups and active-comparator analyses. In drug-specific comparisons with nonuse, the associations were statistically significant for semaglutide and dulaglutide, but not for liraglutide; a tirzepatide-specific mortality estimate was unavailable. Falsification analyses using seizures and pneumonia showed no significant associations [42].

Overall, the direction of the associations generally favored the exposed groups, although several studies reported nonsignificant findings. However, survival was not the primary outcome in all studies, and several reports included multiple secondary, subgroup, sensitivity, or active-comparator analyses. These findings were therefore interpreted according to the hierarchy and context of the original analyses rather than as independent confirmatory results.

### 3.5. Disease Recurrence, Progression and Related Outcomes

Nine studies evaluated outcomes related to cancer recurrence or progression, including incident metastatic disease, disease recurrence or progression, LRR, DFS, PFS, RFS, response-to-therapy status, and NED status among patients in the exposed groups [27,31,38,43,44,46,48,50,51]. Overall, the findings were more heterogeneous than those observed for OS and all-cause mortality.

In colorectal cancer, Dosda et al. reported a lower incidence of metastatic disease among GLP-1 RA users than among patients receiving other non-insulin glucose-lowering therapies [27]. In patients with differentiated thyroid cancer, Patrizio et al. found no significant difference in disease recurrence or structural progression between GLP-1 RA or tirzepatide users and nonusers in a mixed incretin-exposure cohort; response-to-therapy profiles at the last follow-up were also similar between groups. Findings were consistent in sensitivity analyses and analyses stratified by exposure duration [43]. Postoperative GLP-1 RA initiation was associated with longer RFS than DPP-4 inhibitor initiation in Xiang et al.’s cohort of patients with type 2 diabetes who underwent curative-intent resection for hepatocellular carcinoma. RFS was the primary outcome, defined as time to first documented recurrence, with death without prior recurrence treated as a competing event [50].

In lung cancer, Chen Z et al. found no statistically significant differences in PFS for any of the GLP-1 RA treatment-duration categories compared with the untreated group in fully adjusted analyses. Additional spline analyses identified a nonlinear association between treatment duration and progression, although the covariate-adjustment model was not explicitly specified [51]. Following surgery for NSCLC, Pachimatla et al. reported an RFS estimate favoring GLP-1 RA use, but the CI did not support a statistically significant difference between GLP-1 RA users and nonusers. In the advanced NSCLC cohort, GLP-1 RA use was associated with significantly longer PFS during ICI therapy. In this study, RFS and PFS included death as an event [44].

In breast cancer, Tatum et al. observed longer RFS, defined as time to first distant recurrence, with GLP-1 RA or tirzepatide use in mixed incretin-exposure cohorts compared with nonuse and insulin or metformin. The comparison with SGLT2 inhibitors was not significant in the unadjusted matched analysis but reached statistical significance after further adjustment. RFS associations versus nonuse and insulin or metformin persisted in the landmark analyses, while comparisons with SGLT2 inhibitors remained nonsignificant [31]. Sukumar et al. found no statistically significant difference in DFS, defined as recurrence or breast cancer-related death, between GLP-1 RA or tirzepatide users and nonusers in the matched invasive breast cancer cohort [46]. Den et al. reported lower LRR in both mixed incretin-exposure analyses, comparing GLP-1 RA or tirzepatide use, alone or combined with bariatric surgery, with bariatric surgery alone. LRR was approximated using diagnostic codes as proxies for recurrence [38].

Disease status was also evaluated by Chen ST et al., who observed a higher proportion of patients with NED among GLP-1 RA users than among matched nonusers in both the overall and stage-matched cohorts of patients with type 2 diabetes treated with anthracyclines [48].

Overall, no study reported a statistically significant increase in recurrence or progression associated with the exposures evaluated compared with nonuse or an active comparator [27,31,38,43,44,46,48,50,51]. The findings were not uniform across the included cohorts. Detailed outcome data for each study are presented in Table 3.

### 3.6. Supplementary Registry Search

Following screening and deduplication of the ClinicalTrials.gov and WHO ICTRP search results, 31 registered studies were retained for a separate descriptive summary, comprising 28 interventional studies and three observational studies. The registered studies most commonly involved endometrial, breast, and prostate cancer and primarily evaluated semaglutide or tirzepatide. Endometrial cancer studies included protocols assessing pathological response during conservative treatment and changes in tumor proliferation before surgery. In breast cancer, registered endpoints included changes in Ki-67 expression, circulating tumor DNA clearance, and distant disease-free survival. Other studies focused on weight loss, glycemic control, cardiovascular risk, safety, feasibility, or interactions with anticancer treatment. Registry results were posted for one completed retrospective observational study assessing all-cause mortality after cancer diagnosis; for the remaining studies, results were either not posted, or their availability was not reported. Two additional registrations were not included because eligibility could not be confirmed from the available information. Details are presented in Supplementary Table S1. These registrations did not contribute outcome estimates to the main qualitative synthesis of the 26 published studies.

**Table 3:** Outcomes, effect estimates, and main findings of the included studies.

Author, Year	Primary Outcome	Secondary Outcome	Exploratory Outcomes/Analyses	Sensitivity Analyses	Effect Estimate (95% CI)	*p*-Value	Follow-Up *	Main Results
Kohler et al., 2026 [26]	Overall survival (OS), operationalized as all-cause death	NR	NR	OS: 6- and 12-month landmark analyses	Nonuse vs. GLP-1 RA use: main analysis, HR 1.915 (1.770–2.073); 6-month landmark, HR 1.715 (1.570–1.873); 12-month landmark, HR 1.653 (1.496–1.827)	*p* < 0.001 for the main analysis and both landmark analyses	Outcome window: day 1–3650; median follow-up NR	GLP-1 RA users had lower all-cause mortality than nonusers (17.6% vs. 35.0%) and improved OS
Dosda et al., 2026 [27]	All-cause mortality, incident non-digestive cancer and metastatic CRC	Ischemic stroke; myocardial infarction; incident atrial fibrillation; HF hospitalization; composite of all-cause death, ischemic stroke, myocardial infarction, or HF hospitalization; presbyopia (falsification endpoint)	NR	NR	GLP-1 RA use vs. other non-insulin glucose-lowering therapies: all-cause mortality, HR 0.588 (95% CI 0.451–0.766); metastatic CRC, HR 0.608 (95% CI 0.408–0.904); incident non-digestive cancer, HR 0.75 (95% CI 0.57–0.98)	All-cause mortality: *p* < 0.001; metastatic CRC: *p* = 0.01; incident non-digestive cancer: *p* = 0.04.	Median 731 vs. 779 days (GLP-1 RA vs. comparator); maximum 5 years after CRC diagnosis	GLP-1 RA use was associated with lower all-cause mortality (11.5% vs. 20.4%) and lower risks of metastatic CRC and incident non-digestive cancer compared with other non-insulin glucose-lowering therapies.
Wang et al., 2025 [28]	5-year all-cause mortality	NR	All-cause mortality in underpowered subgroups	Exclusion of first-year events; follow-up beyond 5 years; multivariable Cox analysis in unmatched cohorts; 6-, 12-, and 36-month landmark analyses	Mixed incretin-exposure cohort vs. nonuse—main analysis: HR 0.45 (95% CI 0.35–0.59). Adjusted analysis: HR 0.47 (0.37–0.60); first-year exclusion: HR 0.52 (0.37–0.72); extended follow-up: HR 0.46 (0.35–0.60) Landmark analyses: 6 months, HR 0.46 (0.34–0.62); 12 months, HR 0.46 (0.32–0.64); 36 months, HR 0.50 (0.31–0.80). Drug-specific stratified analyses vs. nonuse: semaglutide, HR 0.32 (0.20–0.51); dulaglutide, HR 0.51 (0.32–0.81); liraglutide, HR 0.72 (0.38–1.37); tirzepatide, HR 0.35 (0.10–1.27). Exenatide: not separately analyzed (*N* = 37)	Main analysis (log-rank): *p* < 0.001. Landmark analyses: 6 and 12 months, *p* < 0.0001; 36 months, *p* = 0.003. Drug-specific analyses: semaglutide, *p* < 0.0001; dulaglutide, *p* = 0.004; liraglutide, *p* = 0.32; tirzepatide, *p* = 0.10. Adjusted, first-year-exclusion, and extended-follow-up analyses: *p*-values NR in the available materials.	Median 703 vs. 879 days (mixed incretin exposure vs. nonuse); up to 5 years in the primary analysis	GLP-1 RA or tirzepatide use in the mixed incretin-exposure cohort was associated with lower 5-year all-cause mortality than nonuse (7.94% vs. 19.71%).
Patrizio et al., 2026 [43]	DTC recurrence or progression	Response to therapy at last follow-up	NR	Entire-cohort analysis (*N* = 1072); multistate-model analyses assuming 30 or 180 days in the recurrence/progression state, compared with 90 days in the primary model.	Mixed incretin-exposure cohort vs. nonuse: main survival subcohort (*N* = 840): adjusted HR 0.91 (95% CI 0.65–1.27); entire-cohort sensitivity analysis (*N* = 1072): adjusted HR 0.75 (95% CI 0.54–1.04). Exposure-duration stratified analyses: <12 months, adjusted HR 0.76 (95% CI 0.19–3.12); 12–24 months, adjusted HR 1.25 (95% CI 0.60–2.58); >24 months, adjusted HR 0.80 (95% CI 0.52–1.23)	Main survival subcohort: *p* = 0.57; entire-cohort sensitivity analysis: *p* = 0.07 Response to therapy: *p* = 0.08 Duration-stratified and alternative disease-state-duration analyses: *p*-values NR.	Median 69 months (IQR 28–141), overall cohort; follow-up for the main survival subcohort: NR	GLP-1 RA or tirzepatide exposure (mixed incretin-exposure cohort) was not significantly associated with DTC recurrence, progression, or response to therapy. Drug-specific estimates were unavailable
Chan et al., 2026 [29]	All-cause mortality	Healthcare utilization (hospitalization, ICU admission, and emergency department visits); immune-related and other adverse events	Subgroup analyses of mortality and selected secondary outcomes by demographic and clinical characteristics, cancer type, ICI agent, and concomitant therapies	90-day landmark; Global Network replication; semaglutide-only and combined liraglutide/dulaglutide analyses; additional propensity-score matching incorporating ICI-class indicators; negative-control outcomes. Per-protocol analysis was prespecified, but separate estimates were not reported	All-cause mortality: HR 0.55 (95% CI 0.51–0.61). Mortality sensitivity analyses: Global Network, HR 0.57 (95% CI 0.52–0.63); 90-day landmark, HR 0.56 (95% CI 0.50–0.63); semaglutide-only, HR 0.41 (95% CI 0.34–0.50); combined liraglutide/dulaglutide, HR 0.82 (95% CI 0.68–0.98); additional matching for ICI class, HR 0.72 (95% CI 0.66–0.80).	Primary analysis: *p* < 0.001. Global Network, 90-day landmark, semaglutide-only, and additional ICI-class matching analyses: *p* < 0.001; combined liraglutide/dulaglutide analysis: *p* = 0.028.	Up to 36 months; median follow-up NR	GLP-1 RA use at ICI initiation was associated with a lower 36-month risk of all-cause mortality than no GLP-1 RA use (26.36% vs. 42.77%)
Mahadevan et al., 2026 [30]	Overall survival based on all-cause mortality	All-cause hospitalization, MACE, pneumonia, sepsis, pulmonary embolism, diabetic ketoacidosis and hyperosmolar hyperglycemic state	NR	HbA1c- and obesity-stratified subanalyses	Mixed incretin-exposure cohorts: GLP-1 RA vs. metformin: HR 0.875 (95% CI 0.778–0.985); new-start GLP-1 RA vs. insulin: HR 0.786 (95% CI 0.662–0.934), with background metformin in both new-start groups. Stratified overall-cohort analyses: optimal glycemic control, HR 0.830 (95% CI 0.675–1.020); moderate control, HR 0.821 (95% CI 0.665–1.014); suboptimal control, HR 0.762 (95% CI 0.539–1.077); obesity, HR 0.877 (95% CI 0.759–1.013); nonobese, HR 1.036 (95% CI 0.838–1.279).	GLP-1 RA vs. metformin: *p* = 0.0268; new-start GLP-1 RA vs. insulin: *p* = 0.0062. HbA1c-stratified analyses: *p* = 0.128 for each category; obesity: *p* = 0.156; nonobese: *p* = 0.746	Analysis window: 6 months–2.5 years after systemic anticancer therapy initiation; median follow-up NR	GLP-1 RA or tirzepatide use (mixed incretin-exposure cohort) was associated with lower all-cause mortality than metformin in the overall monotherapy cohort and insulin in the new-start cohort receiving background metformin. Drug-specific estimates were unavailable
Pachimatla et al., 2025 [44]	NR	NR	Postsurgical cohort: RFS and OS; advanced-disease cohort receiving ICIs: PFS and OS	NR	Post-PSM univariable Cox estimates, GLP-1 RA use vs. nonuse: Postsurgical cohort: RFS HR 0.41 (95% CI 0.16–1.04); OS HR 0.78 (95% CI 0.44–1.38); advanced-disease cohort: PFS HR 0.31 (95% CI 0.10–0.94); OS HR 0.41 (95% CI 0.16–1.01)	One-sided *p*-values: Postsurgical RFS: *p* = 0.026; postsurgical OS: *p* = 0.20; advanced-disease PFS: *p* = 0.019; advanced-disease OS: *p* = 0.027	Duration NR; outcomes measured from resection in the postsurgical cohort and ICI initiation in the advanced-disease cohort	Point estimates favored GLP-1 RA use across outcomes; only advanced-cohort PFS had a 95% CI excluding 1
Tatum et al., 2026 [31]	All-cause mortality	RFS	NR	6- and 12-month landmark analyses for all-cause mortality and RFS	Post-PSM HRs (95% CI); mortality first, RFS second. Unadjusted: Nonuse: 0.35 (0.21–0.58); 0.44 (0.30–0.64). Insulin/metformin: 0.09 (0.06–0.15); 0.33 (0.21–0.50). SGLT2i: 0.97 (0.82–1.14); 0.91 (0.71–1.18). Adjusted: Nonuse: 0.42 (0.28–0.62); 0.56 (0.46–0.67). Insulin/metformin: 0.12 (0.08–0.18); 0.44 (0.36–0.54). SGLT2i: 0.77 (0.68–0.88); 0.84 (0.73–0.97). Landmark mortality vs. nonuse: 6 months: 0.68 (0.42–1.11); 12 months: 0.63 (0.37–1.07).	GLP-1 RA vs. nonuse and vs. insulin/metformin: mortality and RFS, *p* < 0.001 in both unadjusted and adjusted analyses. GLP-1 RA vs. SGLT2i: mortality, *p* = 0.72 unadjusted and *p* < 0.001 adjusted; RFS, *p* = 0.46 unadjusted and *p* = 0.02 adjusted; Landmark analyses: *p*-values NR	Up to 10 years. Median follow-up, days (mortality/RFS; exposure vs. comparator): nonuse, 819/806 vs. 931/934; insulin/metformin, 625/625 vs. 525/532; SGLT2i, 806/804 vs. 776/774	GLP-1 RA or tirzepatide use (mixed incretin-exposure cohort) was associated with lower all-cause mortality and distant recurrence compared with nonuse and insulin/metformin. Compared with SGLT2i, the matched unadjusted analyses were nonsignificant, whereas the adjusted Cox models favored GLP-1 RA exposure. Drug-specific estimates were unavailable
Fawzy et al., 2025 [32]	All-cause mortality	NR	NR	NR	Post-PSM, mixed incretin-exposure cohort: HR 0.56 (95% CI 0.49–0.63). Before PSM: HR 0.509 (0.453–0.571). Drug-specific HRs versus nonuse: Semaglutide: 0.27 (0.21–0.35). Liraglutide: 0.75 (0.58–1.03). Dulaglutide: 0.52 (0.43–0.64). Exenatide: 0.90 (0.61–1.32). Tirzepatide: 0.16 (0.09–0.28)	*p* < 0.001 for overall survival comparisons before and after PSM. Drug-specific analyses: *p* < 0.05 for semaglutide, dulaglutide and tirzepatide; liraglutide and exenatide were nonsignificant	Median 523 vs. 664 days; maximum approximately 15 years	GLP-1 RA or tirzepatide use (mixed incretin-exposure cohort) was associated with lower all-cause mortality than nonuse both before and after PSM (11.7% vs. 24.7% after PSM) among patients with NEN and diabetes or obesity.
Cuomo, 2025 [45]	Five-year all-cause mortality, including time to death	Composite cardiovascular outcome (cerebral or myocardial infarction); composite of clinical markers associated with advanced colon cancer (sepsis, abdominal pain, constipation, ascites, anemia, or neoplastic pain). Secondary analyses compared exposed five-year survivors with unexposed patients who died within five years	NR; BMI-stratified analyses were reported without an explicit exploratory designation	CEA-based sensitivity analyses: no CEA adjustment, or adjustment using CEA as a continuous variable, dichotomized at the median, or categorized into tertiles	Five-year mortality: unadjusted OR 0.379 (95% CI 0.21–0.64); fully adjusted OR 0.282 (95% CI 0.13–0.54). Time to death: unadjusted HR 0.732 (95% CI 0.50–1.07); fully adjusted HR 0.816 (95% CI 0.52–1.29). BMI ≥ 35 kg/m^2^, fully adjusted models: five-year mortality, OR 0.051 (0.003–0.26); time to death, HR 0.071 (0.01–0.51). CEA sensitivity analyses, five-year mortality: continuous CEA, OR 0.374 (0.209–0.627); high/low CEA, OR 0.361 (0.202–0.607); CEA tertiles, OR 0.359 (0.201–0.604)	Five-year mortality: *p* < 0.001 unadjusted and fully adjusted. Time to death: *p* = 0.106 unadjusted; *p* = 0.381 fully adjusted. BMI ≥ 35 kg/m^2^, fully adjusted models: five-year mortality, *p* = 0.004; time to death, *p* = 0.009. CEA sensitivity analyses: all *p* < 0.001	5 years; median follow-up NR	GLP-1 RA or tirzepatide use (mixed incretin-exposure cohort) was associated with lower odds of five-year mortality in logistic models, but not with a significant difference in time to death in the overall Cox models. Drug-specific estimates were unavailable
Sukumar et al., 2026 [46]	No single primary outcome specified; evaluated outcomes included weight change, DFS (recurrence or breast cancer-related death) and OS (all-cause mortality)	NR	Weight change and predictors at 6 and 12 months in pooled semaglutide/tirzepatide cohorts treated for ≥6 and ≥12 months, respectively	OS landmark analyses at 6, 12, 24, 36 and 60 months after breast cancer diagnosis, conducted to address potential immortal time bias	Post-PSM (810 users vs. 1620 nonusers): DFS: HR 0.95 (95% CI 0.79–1.14) OS: HR 0.37 (95% CI 0.27–0.53). OS landmark analyses: 6 months: HR 0.38 (0.27–0.53). 12 months: HR 0.39 (0.28–0.55). 24 months: HR 0.43 (0.30–0.62). 36 months: HR 0.47 (0.32–0.69). 60 months: HR 0.50 (0.32–0.76)	DFS: *p* = 0.567 OS: *p* < 0.0001. OS landmark analyses: *p* < 0.0001 at 6, 12, 24 and 36 months; *p* = 0.00146 at 60 months	Overall mixed incretin-exposure cohort (*N* = 1022): median 4.7 years from breast cancer diagnosis; median follow-up for matched cohorts: NR	GLP-1 RA or tirzepatide use (mixed incretin-exposure cohort) was associated with improved OS, but no statistically significant difference in DFS was observed. Drug-specific survival estimates were unavailable
Chiang et al., 2025 [33]	Incident MACE (myocardial infarction, coronary revascularization, heart failure, ischemic stroke or cardiac arrest)	Individual MACE components, myocarditis and pericarditis (secondary efficacy outcomes); all-cause mortality and serious GLP-1 RA-related and immune-related adverse events (safety outcomes)	NR	Active-comparator analyses versus SGLT2i and DPP-4i, excluding concurrent use of the compared drug classes within each comparison	All-cause mortality: Main analysis, post-PSM univariate Cox model: GLP-1 RA vs. other glucose-lowering therapy HR 0.37 (95% CI 0.27–0.50). Active-comparator sensitivity analyses GLP-1 RA vs. SGLT2i HR 0.88 (95% CI 0.53–1.45); GLP-1 RA vs. DPP-4i HR 0.59 (95% CI 0.39–0.88)	GLP-1 RA vs. other glucose-lowering therapy: *p* < 0.001 (log-rank); GLP-1 RA vs. SGLT2i: *p* = 0.607; GLP-1 RA vs. DPP-4i: *p* = 0.009	Median 12 months from ICI initiation	GLP-1 RA or tirzepatide use (mixed incretin-exposure cohort) was associated with lower all-cause mortality than other glucose-lowering therapy (12.4% vs. 29.9%). Drug-specific estimates were unavailable
Radwan et al., 2025 [49]	All-cause mortality	NR	NR	Assessment of potential unmeasured confounding using E-values: 1.21 versus SGLT2i and 2.73 versus DPP-4i	Post-PSM Cox models under an intention-to-treat framework: GLP-1 RA vs. SGLT2i: HR 1.03 (0.85–1.23). GLP-1 RA vs. DPP-4i: HR 0.60 (0.51–0.70)	Exact *p*-values NR; GLP-1 RA vs. SGLT2i: NS; GLP-1 RA vs. DPP-4i: statistically significant	From first prescription (index date): GLP-1 RA vs. SGLT2i: median 1.65 years (IQR 0.76–2.76). GLP-1 RA vs. DPP-4i: median 1.73 years (IQR 0.83–2.79).	No statistically significant difference in all-cause mortality was observed between GLP-1 RA and SGLT2i users (8.7% vs. 8.6%), but mortality was significantly lower with GLP-1 RA than with DPP-4i (9.1% vs. 15.1%)
Nelson et al., 2026 [47]	Overall survival based on all-cause mortality	NR	NR	Analysis restricted to patients with stage I–III breast cancer, excluding stage IV disease	Main analysis (prespecified 2-year landmark): adjusted HR 0.51 (95% CI 0.28–0.93). Sensitivity analysis excluding stage IV disease: adjusted HR 0.45 (95% CI 0.23–0.85)	Main analysis: *p* = 0.03. Sensitivity analysis: *p* = 0.01. Kaplan–Meier comparisons: log-rank *p* = 0.02 overall and *p* = 0.006 in stage I–III patients	Median follow-up from breast cancer diagnosis: 6.9 vs. 6.8 years	GLP-1 RA use was associated with lower all-cause mortality than nonuse (7.5% vs. 13.3%; stage I–III sensitivity analysis: 6.5% vs. 13.1%).
Javaid et al., 2026 [34]	CTRCD (new-onset cardiomyopathy, HF or intravenous loop diuretic initiation)	Incident HF, acute myocardial infarction, atrial fibrillation/flutter, hospitalization or ED visits and all-cause mortality	NR; subgroup analyses examined CTRCD by antineoplastic therapy class and clinical outcomes in metastatic cancer, without an explicit exploratory designation	Assessment of unmeasured confounding using E-values; all-cause mortality E-values: 5.90 at 1 year and 4.70 at 3 years	All-cause mortality: 1-year HR 0.31 (95% CI 0.29–0.34); 3-year HR 0.38 (95% CI 0.35–0.40). Metastatic cancer subgroup: 1-year HR 0.35 (95% CI 0.29–0.42); 3-year HR 0.39 (95% CI 0.34–0.46)	*p* < 0.01 for both overall and metastatic subgroup estimates	Outcomes assessed at 1 and 3 years after the index event; median follow-up NR	GLP-1 RA use was associated with lower all-cause mortality than nonuse at 1 and 3 years
Scalia et al., 2025 [35]	No single primary outcome was explicitly designated; evaluated outcomes included all-cause mortality, all-cause hospitalization, acute HF, acute renal failure/new RRT, new-onset atrial fibrillation/flutter and cardiac arrest	NR	NR	NR	All-cause mortality: aOR 0.592 (95% CI 0.337–1.041); HR 0.653 (95% CI 0.387–1.101)	All-cause mortality: log-rank *p* = 0.107; aOR *p*-value: NR	Mean ± SD: 295.4 ± 109.6 vs. 305.9 ± 102.0 days; outcomes assessed through 1 year.	No statistically significant difference in all-cause mortality was observed between GLP-1 RA or tirzepatide users (mixed incretin-exposure cohort) and nonusers (11.4% vs. 17.9%). Drug-specific mortality estimates were unavailable
Vignarajah et al., 2025 [36]	No single primary outcome specified; evaluated outcomes included all-cause mortality, HF exacerbation, all-cause hospitalization or ED visits, atrial fibrillation/flutter and ventricular tachycardia	NR	NR	E-value analysis for unmeasured confounding (all-cause mortality: 2.90); gastroenteritis falsification analysis (*p* = 0.72)	All-cause mortality: HR 0.57 (95% CI 0.43–0.77)	*p* < 0.001	1-year follow-up; median follow-up NR	GLP-1 RA use was associated with lower 1-year all-cause mortality than nonuse (8.4% vs. 14.3%)
Cornelio et al., 2026 [37]	Five-year incidence of MACE, comprising myocardial infarction, ischemic stroke, cardiac arrest, coronary revascularization or heart failure	Ischemic heart disease, myocardial infarction, ischemic stroke, cardiac arrest, heart failure, end-stage renal disease and all-cause mortality. Safety outcomes: pancreatitis and cholelithiasis.	Renal outcome analysis (ESRD, including dialysis)	NR	All-cause mortality: RR 0.384 (95% CI 0.359–0.411)	All-cause mortality: *p* < 0.001	Up to 5 years after the index date; actual follow-up NR	GLP-1 RA or tirzepatide use (mixed incretin-exposure cohort) was associated with lower five-year all-cause mortality than other glucose-lowering therapy (5.4% vs. 14.0%). Drug-specific mortality estimates were unavailable
Den et al., 2026 [38]	No single primary outcome was explicitly designated; evaluated outcomes included OS (all-cause mortality) and LRR.	NR	NR	NR	Study 1 (GLP-1 RA or tirzepatide use vs. bariatric surgery alone): mortality HR 0.57 (95% CI 0.45–0.73); LRR HR 0.52 (95% CI 0.39–0.70); Study 2 (GLP-1 RA or tirzepatide use combined with bariatric surgery vs. bariatric surgery alone): mortality HR 0.44 (95% CI 0.29–0.67); LRR HR 0.52 (95% CI 0.33–0.81)	OS: log-rank *p* < 0.0001 for both studies; LRR: exact *p*-values NR; statistically significant based on the 95% CIs in both studies	Median follow-up: Study 1, 590 days (GLP-1 RA or tirzepatide) vs. 1408 days (bariatric surgery); Study 2, 645 days (combination therapy) vs. 1314 days (bariatric surgery alone); Outcome window: 30 days to 10 years after the index event	Mixed incretin-exposure cohorts. Study 1 (drug therapy vs. bariatric surgery): observed mortality, 2.5% vs. 8.4%; LRR, 1.8% vs. 4.7%; estimated 10-year OS, 87% vs. 83%. Study 2 (drug therapy plus bariatric surgery vs. bariatric surgery alone): observed mortality, 2.6% vs. 10.0%; LRR, 2.5% vs. 5.8%; estimated 10-year OS, 91% vs. 80%. Drug-specific estimates were unavailable
Xiang et al., 2026 [50]	RFS, defined as time to first documented recurrence, with death without recurrence treated as a competing event	OS, defined as time to all-cause death	Prespecified subgroup analyses (ITT: RFS and OS; PP: RFS only), described as exploratory in the Discussion. Post hoc ITT analyses included 3- and 6-month persistence landmarks and comparisons of short-acting and long-acting GLP-1 RAs with DPP-4 inhibitors	Prespecified PP analysis; alternative treatment-weight truncation thresholds, overlap weighting and 1:1 PSM; alternative censoring-weight caps in PP analyses; a prespecified 3-month landmark; 24-month time-split models; competing-risk and RMST analyses; positive and negative outcome controls and E-value assessments	Weighted ITT analysis RFS: csHR 0.80 (95% CI 0.67–0.96) OS: HR 0.58 (95% CI 0.47–0.71). Prespecified PP sensitivity analysis: RFS: csHR 0.35 (95% CI 0.25–0.49); OS: HR 0.27 (95% CI 0.11–0.68). PP RMST differences (GLP-1 RA minus DPP-4i): RFS at 36 months, +2.92 months (95% CI 1.81–3.85); OS at 60 months, +3.02 months (95% CI 1.52–4.15).	Weighted ITT: RFS: *p* = 0.016 OS: *p* < 0.001. PP: RFS: *p* < 0.001; OS: *p* = 0.005. PP RMST differences: *p* < 0.001 for both outcomes	Median 50.8 months (IQR 33.6–63.9), from the first qualifying postoperative prescription until the relevant outcome, loss to follow-up or 1 October 2025.	GLP-1 RA initiation was associated with longer RFS and OS than DPP-4i initiation in the weighted ITT analysis. In exploratory analyses, neither the RFS nor the OS estimate for long-acting GLP-1 RAs reached statistical significance
Chen ST et al., 2026 [48]	Five-year all-cause mortality	Cancer-related and CVD-related mortality, CVD/HF hospitalization and change in LVEF. Additional reported oncologic outcome, without an explicit primary/secondary designation: NED.	NR	Additional restricted-cohort analyses, not explicitly designated as sensitivity analyses: cancer-stage matching and restriction to ≥3 months of GLP-1 RA use before anthracycline initiation	Overall cohort: all-cause mortality HR 0.54 (95% CI 0.31–0.92), adjusted HR 0.48 (0.28–0.82); cancer-related mortality HR 0.47 (0.24–0.92) Stage-matched cohort: all-cause mortality HR 0.46 (0.24–0.87), adjusted HR 0.39 (0.20–0.75); cancer-related mortality HR 0.42 (0.19–0.91) ≥3-month pre-anthracycline subgroup (2.5-year outcomes): all-cause mortality HR 0.46 (0.21–0.99), adjusted HR 0.47 (0.18–1.20); cancer-related mortality HR 0.56 (0.22–1.41)	Overall cohort: all-cause mortality *p* = 0.03, adjusted *p* = 0.007; cancer-related mortality *p* = 0.03; NED *p* = 0.01 Stage-matched cohort: all-cause mortality *p* = 0.007, adjusted *p* = 0.002; cancer-related mortality *p* = 0.02; NED *p* = 0.007 ≥3-month pre-anthracycline subgroup (2.5-year outcomes): all-cause mortality *p* = 0.049, adjusted *p* = 0.08; cancer-related mortality *p* = 0.23	Outcome window: 5 years; ≥3-month pre-anthracycline subgroup: 2.5 years; follow-up began at anthracycline initiation; median follow-up NR	GLP-1 RA users had lower all-cause and cancer-related mortality in the overall and stage-matched cohorts. NED was more frequent than among matched nonusers (overall cohort: 58.9% vs. 39.7%; stage-matched cohort: 60.9% vs. 38.0%)
Chen Z et al., 2026 [51]	Progression-free survival (time from treatment initiation to progression or death) and overall survival (time from treatment initiation to all-cause death)	NR	NR	Analyses repeated with users redefined as receiving ≥ 14 continuous days of treatment instead of ≥28 days; mortality-duration spline analysis	Fully adjusted model (Model 4), versus the study-defined untreated reference group: PFS: <106 days HR 0.51 (95% CI 0.23–1.13); 106–292 days HR 0.89 (95% CI 0.53–1.50); 293–559 days HR 0.50 (95% CI 0.22–1.12); ≥560 days HR 0.43 (95% CI 0.18–1.03) OS: <106 days HR 0.42 (95% CI 0.16–1.12); 106–292 days HR 0.86 (95% CI 0.48–1.54); 293–559 days HR 0.52 (95% CI 0.21–1.25); ≥560 days HR 0.44 (95% CI 0.16–1.18)	Duration-category estimates: NS (all fully adjusted 95% CIs included 1). Spline analyses: overall association *p* < 0.001 for both progression and mortality; nonlinearity *p* = 0.031 for progression and *p* = 0.086 for mortality	Median follow-up NR	In fully adjusted analyses, all point estimates favored GLP-1 RA use versus the study-defined untreated group, but none of the quartile-specific associations with PFS or OS was statistically significant.
Lin et al., 2026 [39]	Wasting-related diagnosis composite: sarcopenia, cachexia or malnutrition	Contextual secondary endpoints: all-cause mortality and immune-related adverse events; key secondary endpoints: acute-care utilization and related safety outcomes; additional selected safety outcomes	All-cause mortality interpreted as exploratory; subgroup and interaction analyses; exploratory mortality E-values; descriptive longitudinal body-weight and blood-cell analyses	Per-protocol analysis; 3-month landmark analysis; 12-month washout; replication in the Global Network; semaglutide-restricted analysis; separate active-comparator OS analysis versus metformin or sulfonylurea; separate primary-comparator OS analysis; negative-control analyses.	All-cause mortality, primary cohort (mixed incretin-exposure cohort): HR 0.41 (95% CI 0.28–0.61); 12-month washout: HR 0.26 (95% CI 0.15–0.44); 3-month landmark: HR 0.23 (95% CI 0.12–0.44); Global Network: HR 0.35 (95% CI 0.21–0.58); active-comparator OS: HR NR. Semaglutide-restricted mortality estimate: NA.	All-cause mortality: *p* < 0.001 (also < 0.001 after Benjamini–Hochberg adjustment); 12-month washout and Global Network mortality: *p* < 0.001; 3-month landmark mortality: *p* NR; active-comparator OS: log-rank *p* = 0.003	Up to 36 months from day 1 after ICI initiation; median follow-up NR	GLP-1 RA or tirzepatide use (mixed incretin-exposure cohort) was associated with lower all-cause mortality than nonuse (3.88% vs. 9.92%). Drug-specific mortality estimates were unavailable
Wu et al., 2026 [40]	CTRCD: new cardiomyopathy/HF, newly documented LVEF ≤ 40% or initiation of intravenous diuretic therapy	All-cause mortality; all-cause hospitalization; ASCVD; arrhythmia; thrombotic disorders; HF hospitalization; composite cardiovascular outcomes	NR	CTRCD: Pretreatment-restricted analysis; landmark/time-window analyses (1 day–6 months, 1 month–1 year and 6 months–1 year); era-restricted analyses (2017–2019, 2020–2025 and ≥2022); negative-control outcomes (skin cancer and traumatic brain injury); E-value analysis	All-cause mortality: HR 0.68 (95% CI 0.61–0.75)	*p* < 0.001	Maximum 1 year; median follow-up: 365 (IQR 251–365) vs. 365 (IQR 340–365) days	GLP-1 RA use was associated with lower all-cause mortality than nonuse (7.1 vs. 10.9 deaths per 100 person-years)
Kwon et al., 2026 [41]	Ninety-day postoperative complications after partial nephrectomy	Not explicitly specified; OS also evaluated	NR	Restriction to patients treated during 2020–2025 for 90-day postoperative complications; no OS sensitivity estimate reported	Overall survival: adjusted HR 0.665 (95% CI 0.488–0.907)	Adjusted OS: *p* = 0.01; 2-year OS: log-rank *p* = 0.002; 3-year OS: log-rank *p* = 0.001.	Median follow-up 665 vs. 1161 days, Kaplan–Meier OS analyses at 2 and 3 years after partial nephrectomy.	GLP-1 RA use was associated with improved overall survival compared with nonuse: Kaplan–Meier OS estimates were 97.28% vs. 94.29% at 2 years and 96.10% vs. 91.96% at 3 years
Chi et al., 2026 [42]	All-cause mortality up to 3 years after the first recorded brain metastasis diagnosis	NR	NR	Falsification analyses using seizures and pneumonia; no separately labelled sensitivity analyses reported	All-cause mortality, overall mixed incretin-exposure cohort versus nonuse: HR 0.63 (95% CI 0.54–0.72). Drug-type subgroup analyses versus nonuse: semaglutide: HR 0.38 (0.29–0.49); dulaglutide: HR 0.75 (0.61–0.93); liraglutide: HR 0.96 (0.71–1.31); tirzepatide: NA (<10 deaths in each group). Active-comparator analyses: versus DPP-4 inhibitors: HR 0.62 (0.52–0.74); SGLT2 inhibitors: HR 0.68 (0.52–0.90); sulfonylureas: HR 0.62 (0.52–0.73); thiazolidinediones: HR 0.62 (0.50–0.76); insulin, HR 0.58 (0.49–0.67).	Overall mortality: *p* < 0.001. Drug-type subgroup and active-comparator analyses: exact *p*-values NR.	Up to 3 years; median follow-up NR	GLP-1 RA or tirzepatide use (mixed incretin-exposure cohort) was associated with lower 3-year all-cause mortality than nonuse (37.3% vs. 54.8%)

* Unless otherwise stated, follow-up values are presented for the exposed group followed by the comparator group. aOR, adjusted odds ratio; ASCVD, atherosclerotic cardiovascular disease; BMI, body mass index; CEA, carcinoembryonic antigen; CI, confidence interval; CRC, colorectal cancer; csHR, cause-specific hazard ratio; CTRCD, cancer therapy-related cardiac dysfunction; CVD, cardiovascular disease; DFS, disease-free survival; DPP-4i, dipeptidyl peptidase-4 inhibitor; DTC, differentiated thyroid cancer; ED, emergency department; ESRD, end-stage renal disease; GLP-1 RA, glucagon-like peptide-1 receptor agonist; HbA1c, glycated hemoglobin; HF, heart failure; HR, hazard ratio; ICI, immune checkpoint inhibitor; ICU, intensive care unit; IQR, interquartile range; ITT, intention-to-treat; LRR, locoregional recurrence; LVEF, left ventricular ejection fraction; MACE, major adverse cardiovascular events; *N*, sample size; NA, not available; NED, no evidence of disease; NEN, neuroendocrine neoplasm; NR, not reported; NS, not statistically significant; OR, odds ratio; OS, overall survival; PFS, progression-free survival; PP, per-protocol; PSM, propensity score matching; RFS, recurrence-free survival; RMST, restricted mean survival time; RR, risk ratio; RRT, renal replacement therapy; SD, standard deviation; SGLT2i, sodium-glucose cotransporter 2 inhibitor; vs., versus.

## 4. Discussion

This systematic review included 26 retrospective cohort studies evaluating the association between GLP-1 RA use, including mixed incretin exposure in some cohorts, and oncologic or survival outcomes in patients with cancer. Twenty-five studies evaluated OS and/or all-cause mortality, while nine reported outcomes related to cancer recurrence or progression. Most mortality and survival estimates favored the exposed groups, although several findings were nonsignificant.

The most consistent finding across the included studies was the association between GLP-1 RA use or mixed incretin exposure and lower mortality or longer survival. Favorable associations were reported across several site-specific and mixed-cancer cohorts, although OS findings in lung cancer were inconclusive and some active comparator analyses were not significant [31,33,44,49,51]. This pattern is clinically relevant because GLP-1 RAs are increasingly used in patients with obesity and type 2 diabetes, including those with cancer [18].

These findings provide some hope regarding the use of GLP-1 RAs in patients with cancer and obesity or type 2 diabetes. However, they do not establish oncologic safety or a causal benefit because all included studies were observational.

Findings regarding recurrence and progression were more limited and less consistent than those for survival. Favorable associations were reported in some cohorts, whereas other studies found no significant differences [27,31,38,43,44,46,48,50,51]. Although no study reported a significantly higher risk in the exposed groups, the limited number of studies and variation in outcome definitions prevent firm conclusions. The more consistent associations with all-cause mortality may partly reflect benefits unrelated to direct tumor control, including the established cardiovascular benefits of GLP-1 RAs [52]. This may be particularly relevant because several included studies involved patients receiving potentially cardiotoxic cancer treatments [34,35,36,40,48].

Evidence on cancer incidence provides additional, although indirect, context. In a large retrospective cohort of patients with type 2 diabetes, Wang L et al. found that GLP-1 RA use was associated with a lower risk of several obesity-associated cancers compared with insulin, but no significant risk reduction compared with metformin. Kidney cancer risk was higher in comparison with metformin [53]. In the safety analysis of the randomized Semaglutide Effects on Cardiovascular Outcomes in People with Overweight or Obesity (SELECT) trial, Kushner et al. reported similar frequencies of malignant neoplasms with semaglutide and placebo among patients with overweight or obesity and established cardiovascular disease without diabetes [54]. However, these findings do not establish whether GLP-1 RAs affect recurrence, progression or survival in patients with an established malignancy.

Several mechanisms may contribute to the observed associations. In addition to promoting weight loss and improving glycemic control and insulin sensitivity, GLP-1 RAs may reduce systemic inflammation and alter metabolic conditions that can support tumor growth [7,18,21,55]. Preclinical studies have also reported effects on the phosphoinositide 3-kinase/protein kinase B/mechanistic target of rapamycin (PI3K/Akt/mTOR), extracellular signal-regulated kinase/mitogen-activated protein kinase (ERK/MAPK), adenosine monophosphate-activated protein kinase (AMPK), and nuclear factor kappa B (NF-κB) pathways, together with reduced proliferation, migration, and invasion and increased apoptosis in some tumor models [18,19,56,57]. However, these effects have not been consistent across experimental settings and may vary according to receptor expression, tumor type, drug, dose, and cellular context [19,21,58]. Accordingly, the mechanistic evidence should be considered hypothesis-generating and does not establish that the survival associations observed in clinical cohorts are mediated by direct effects of GLP-1 receptor signaling on tumor biology.

A strength of this review was its focus on outcomes in patients with an established cancer diagnosis, rather than on cancer incidence. The inclusion of both nonuser and active-comparator studies provided a broader and more clinically relevant view of the available evidence. By bringing together survival, recurrence, and progression outcomes across several cancer types, we considered GLP-1 RA use in the clinical context in which these drugs are prescribed in current practice.

An important limitation of the available evidence is the possibility of residual confounding. Although the included studies used matching, weighting, or multivariable adjustment, GLP-1 RA users may still have differed from comparator groups in cancer stage, anticancer treatment, metabolic control, treatment adherence, lifestyle, healthcare use, or access to care. These differences may have independently influenced survival. Confounding by indication and treatment-selection bias are particularly important concerns. Patients receiving GLP-1 RAs may differ systematically from nonusers or active-comparator groups in disease severity, comorbidity burden, metabolic status, healthcare utilization, and other prognostic characteristics. Moreover, patients with advanced cancer, poor performance status, progressive weight loss, or limited life expectancy may be less likely to initiate or continue GLP-1 RA therapy. Such differences could preferentially place patients with a better underlying prognosis in the exposed group and thereby contribute to an apparent survival advantage. Reverse causation therefore cannot be excluded. Although matching, weighting, and multivariable adjustment were used in the included studies, these approaches cannot fully account for unmeasured or incompletely measured confounding.

Descriptively, associations were less consistent in active-comparator analyses, particularly when SGLT2 inhibitors were used as comparators, than in comparisons with nonusers [31,33,49]. This pattern suggests that comparator choice and treatment selection may have influenced the observed estimates, although it does not establish residual confounding or channeling bias. Therefore, the favorable associations cannot be interpreted as evidence that GLP-1 RA use directly improves cancer outcomes.

Other limitations of the available evidence should also be considered. The retrospective designs of the studies and their reliance on routinely collected data made them susceptible to selection bias and exposure or outcome misclassification. The studies differed in cancer type, population, exposure and comparator groups, outcome definitions, follow-up duration and analytical approach. The definition and timing of GLP-1 RA exposure also varied substantially across studies. Exposure was defined using approaches ranging from a single prescription or short treatment period to peri-diagnostic use, post-diagnostic initiation, concurrent use with anticancer therapy, and duration-based exposure categories. These definitions do not represent clinically equivalent exposures and may capture different treatment patterns, treatment durations, and patient populations, thereby limiting direct comparisons across studies. The use of different effect measures also limited direct comparisons between the reported estimates, because HRs, ORs and RRs are not directly equivalent. Only one study reported cancer-related mortality, whereas most evaluated OS or all-cause mortality; therefore, the observed survival differences may partly reflect differences in mortality from non-cancer causes. Cancer stage, tumor characteristics, anticancer treatment details, and information on GLP-1 RA dose, duration and adherence were often unavailable. Follow-up was short, unequal, or incompletely reported in several cohorts and time-related biases could not always be excluded. Finally, because 17 studies used the TriNetX network, some overlap between study populations is possible, particularly among studies with similar populations and study periods. The extent of any overlap could not be determined from the published reports, and the 26 included studies should therefore not necessarily be interpreted as representing 26 fully independent estimates.

In addition, thirteen studies combined selective GLP-1 RAs with dual GLP-1/GIP receptor agonists within the exposure groups defined by the original study authors. These agents are not pharmacologically identical, and drug-specific estimates were available in some, but not all, of these studies. Findings based on combined-exposure estimates cannot be attributed specifically to selective GLP-1 RAs.

Another limitation is the multiplicity of analyses reported across the included studies. Many cohorts evaluated multiple outcomes, comparator groups, exposure definitions, subgroups, and sensitivity analyses, and adjustment for multiple testing was not consistently reported. Consequently, some statistically significant findings may have arisen by chance, particularly among secondary or exploratory analyses. We therefore placed greater emphasis on prespecified or primary analyses, effect estimates and confidence intervals, and consistency across clinically comparable analyses rather than on statistical significance alone.

The present review also had methodological limitations. The search was limited to English-language publications from the previous 10 years and to three electronic databases, so relevant studies published in other languages or indexed elsewhere may have been missed. The exclusion of conference abstracts and posters may also have resulted in the omission of recent findings that were not yet available as full-text articles.

From a clinical perspective, the available evidence did not indicate worse oncologic outcomes in the exposed groups evaluated. However, the evidence is not sufficient to recommend GLP-1 RAs for improving cancer outcomes or to guide treatment based on a possible anticancer effect. Decisions regarding their use should remain based on the management of obesity or diabetes and the individual clinical context, including nutritional status and the risk of muscle loss or cachexia [23].

Important knowledge gaps remain. Prospective cohort studies and randomized controlled trials are needed to determine whether the observed associations represent causal effects rather than residual confounding. Future studies should prioritize cancer-specific mortality, recurrence, and progression outcomes, use standardized outcome definitions, and provide longer and more complete follow-up. Detailed information on cancer stage, anticancer treatment, and medication adherence should also be collected. Further research should assess whether outcomes differ by individual GLP-1 RA, dose, treatment duration, cumulative exposure and cancer type.

## 5. Conclusions

This systematic review found favorable survival associations with GLP-1 RA use or mixed incretin exposure across diverse cancer populations, but the magnitude and statistical consistency of these associations varied substantially by comparator and analytical approach. Findings based on pooled estimates combining selective GLP-1 RAs and dual GLP-1/GIP receptor agonists cannot be attributed specifically to selective GLP-1 RAs. Associations were generally less consistent in active-comparator analyses than in comparisons with nonusers, suggesting that treatment selection and residual confounding may have contributed to the apparent survival benefit. Some statistically significant findings may have arisen by chance, particularly among secondary or exploratory analyses. Evidence regarding cancer recurrence and progression was limited and heterogeneous; although no clear increase in risk was observed, a clinically relevant risk cannot be excluded. These observational findings do not establish a causal treatment effect or support the use of GLP-1 RAs as anticancer therapy. GLP-1 RAs should therefore continue to be prescribed for established indications, such as obesity or type 2 diabetes. Future studies should use well-defined treatment windows, active comparators, cancer-specific outcomes, and longer follow-up.

## Data Availability

No new data were created or analyzed in this study. Data sharing is not applicable to this article.

## Supplementary Materials

The following supporting information can be downloaded at: https://www.mdpi.com/article/10.3390/ijms27209251/s1.

## Abbreviations

The following abbreviations are used in this manuscript:

aOR	adjusted odds ratio
Akt	protein kinase B
AMPK	AMP-activated protein kinase
ASCVD	atherosclerotic cardiovascular disease
BMI	body mass index
CEA	carcinoembryonic antigen
CI	confidence interval
CRC	colorectal cancer
csHR	cause-specific hazard ratio
CTRCD	cancer therapy-related cardiac dysfunction
CVD	cardiovascular disease
DCIS	ductal carcinoma in situ
DM	diabetes mellitus
DFS	disease-free survival
DNA	deoxyribonucleic acid
DPP-4	dipeptidyl peptidase-4
DPP-4i	dipeptidyl peptidase-4 inhibitor
DTC	differentiated thyroid cancer
ED	emergency department
EMR	electronic medical record
ERK	extracellular signal-regulated kinase
ESRD	end-stage renal disease
GIP	glucose-dependent insulinotropic polypeptide
GLP-1 RA	glucagon-like peptide-1 receptor agonist
GLP-1/GIP RA	glucagon-like peptide-1/glucose-dependent insulinotropic polypeptide receptor agonist
HbA1c	glycated hemoglobin
HCC	hepatocellular carcinoma
HF	heart failure
HR	hazard ratio
IARC	International Agency for Research on Cancer
ICI	immune checkpoint inhibitor
ICU	intensive care unit
IGF-1	insulin-like growth factor 1
IL-1β	interleukin-1 beta
IL-6	interleukin-6
IQR	interquartile range
ITT	intention-to-treat
LRR	locoregional recurrence
LVEF	left ventricular ejection fraction
MACE	major adverse cardiovascular events
MAPK	mitogen-activated protein kinase
MeSH	Medical Subject Headings
mTOR	mechanistic target of rapamycin
N	total sample size
NA	not available
NED	no evidence of disease
NEC	neuroendocrine carcinoma
NEN	neuroendocrine neoplasm
NET	neuroendocrine tumor
NF-κB	nuclear factor kappa B
NK	natural killer
NOS	Newcastle–Ottawa Scale
NR	not reported
NS	not statistically significant
NSCLC	non-small cell lung cancer
OR	odds ratio
OS	overall survival
PFS	progression-free survival
PI3K	phosphoinositide 3-kinase
PP	per-protocol
PRISMA	Preferred Reporting Items for Systematic Reviews and Meta-Analyses
PSM	propensity score matching
R0	complete resection with no residual tumor
RCS	restricted cubic spline
RFS	recurrence-free survival
RMST	restricted mean survival time
RR	risk ratio
RRT	renal replacement therapy
SD	standard deviation
SGLT2	sodium-glucose cotransporter 2
SGLT2i	sodium-glucose cotransporter 2 inhibitor
T2D	type 2 diabetes
TNF-α	tumor necrosis factor alpha
TTE	target trial emulation
UCHDW	University of California Health Data Warehouse
vs	versus
WHO ICTRP	World Health Organization International Clinical Trials Registry Platform

## Appendix A

08.08.2026: PUBMED: ((“Glucagon-Like Peptide-1 Receptor Agonists”[Mesh] OR semaglutide[tiab] OR liraglutide[tiab] OR dulaglutide[tiab] OR exenatide[tiab] OR lixisenatide[tiab] OR tirzepatide[tiab] OR “glucagon-like peptide-1 receptor agonists”[tiab] OR “glucagon like peptide 1 receptor agonist”[tiab] OR “glucagon-like peptide-1 agonist”[tiab] OR “glucagon like peptide 1 agonists”[tiab] OR “GLP-1 receptor agonist”[tiab] OR “GLP-1 receptor agonists”[tiab] OR “GLP1 receptor agonists”[tiab] OR “GLP-1 agonist”[tiab] OR “GLP-1 agonists”[tiab] OR “GLP1 agonists”[tiab] OR “GLP-1 RA”[tiab] OR “GLP-1 RAs”[tiab] OR GLP1RA[tiab] OR GLP1RAs[tiab] OR GLP1a[tiab] OR “GLP-1/GIP receptor agonist”[tiab] OR “GLP-1/GIP co-agonist”[tiab]) AND (“Neoplasms”[Mesh] OR cancer*[tiab] OR neoplasm*[tiab] OR malignan*[tiab] OR tumor*[tiab] OR tumour*[tiab] OR carcinoma*[tiab] OR metastas*[tiab] OR oncolog*[tiab]) AND (surviv*[tiab] OR mortalit*[tiab] OR death*[tiab] OR recurren*[tiab] OR relaps*[tiab] OR progress*[tiab])) AND (“2016/01/01”[dp]: “2026/08/08”[dp]) AND english[lang]

08.08.2026: Scopus TITLE-ABS(semaglutide OR liraglutide OR dulaglutide OR exenatide OR lixisenatide OR albiglutide OR tirzepatide OR “glucagon-like peptide-1 receptor agonist” OR “glucagon like peptide 1 agonists” OR “GLP-1 receptor agonist” OR “GLP1 receptor agonist” OR “GLP1 receptor agonists” OR “GLP-1 agonist” OR “GLP1 agonists” OR “GLP-1 RA” OR “GLP-1 RAs” OR “GLP-1RA” OR “GLP-1RAs” OR GLP1RAs OR “GLP-1/GIP receptor agonists”) AND TITLE-ABS-KEY(cancer* OR neoplasm* OR malignan* OR tumor* OR carcinoma* OR metastas* OR oncolog*) AND TITLE-ABS(surviv* OR mortalit* OR death* OR recurren* OR relaps* OR progress*) AND PUBYEAR AFT 2015 AND PUBYEAR BEF 2027 AND LANGUAGE(english)

08.08.2026: WoS** **((TI = (semaglutide OR liraglutide OR dulaglutide OR exenatide OR lixisenatide OR albiglutide OR tirzepatide OR “glucagon-like peptide-1 receptor agonist” OR “glucagon like peptide 1 receptor agonists” OR “glucagon-like peptide-1 agonist” OR “glucagon-like peptide-1 agonists” OR “GLP-1 receptor agonist” OR “GLP-1 receptor agonists” OR “GLP1 receptor agonist” OR “GLP1 receptor agonists” OR “GLP-1 agonist” OR “GLP-1 agonists” OR “GLP-1 RA” OR “GLP-1 RAs” OR “GLP-1RA”) OR AB = (semaglutide OR liraglutide OR dulaglutide OR exenatide OR lixisenatide OR albiglutide OR tirzepatide OR “glucagon-like peptide-1 receptor agonists” OR “glucagon like peptide 1 receptor agonist” OR “glucagon like peptide 1 agonist” OR “glucagon like peptide 1 agonists” OR “GLP-1 receptor agonist” OR “GLP-1 receptor agonists” OR “GLP1 receptor agonists” OR “GLP-1 agonist” OR “GLP-1 agonists” OR “GLP1 agonist” OR “GLP1 agonists” OR “GLP-1 RA” OR “GLP-1 RAs” OR “GLP-1RAs”))

AND TS = (cancer* OR neoplasm* OR malignan* OR tumor* OR tumour* OR carcinoma* OR metastas* OR oncolog*) AND (TI = (surviv* OR mortalit* OR death* OR recurren* OR relaps* OR progress*) OR AB = (surviv* OR mortalit* OR death* OR recurren* OR relaps* OR progress*)) AND PY = (2016–2026) AND LA = (English)

09.10.2026: ClinicalTrials.gov** **AREA[ConditionSearch](cancer OR neoplasm OR carcinoma OR malignancy OR tumor) AND AREA[InterventionSearch](semaglutide OR liraglutide OR dulaglutide OR exenatide OR lixisenatide OR albiglutide OR tirzepatide OR “GLP-1 receptor agonist” OR “GLP-1 RA” OR “glucagon-like peptide-1”)

09.10.2026: WHO ICTRP** **(cancer OR neoplasm* OR carcinoma* OR malignan* OR tumor* OR tumour*) AND (semaglutide OR liraglutide OR dulaglutide OR exenatide OR lixisenatide OR albiglutide OR tirzepatide OR “GLP-1” OR GLP1 OR “glucagon-like peptide”)
