# Supplementary material for: GLP-1 Receptor Agonist Therapy in Patients with Cancer: A Review of Clinical Outcomes
Source: Int J Mol Sci. 2026 Oct 18;27(20):9251. doi: 10.3390/ijms27209251 (PMC13705413; doi:10.3390/ijms27209251)
Supplement: Supplementary file 1 [file ijms-27-09251-s001.zip › ijms-4595859-supplementary.pdf]

Supplementary Table S1. Registered studies evaluating GLP-1–based therapies in adults with a cancer diagnosis

REGISTRY / TRIAL ID	STUDY TITLE	COUNTRY / REGION	CANCER TYPE	STUDY DESIGN	GLP-1–BASED INTERVENTION	COMPARATOR	PRIMARY ENDPOINT(S)	RECRUITMENT STATUS	ESTIMATED/ACTUAL COMPLETION DATE	RESULTS AVAILABLE
ClinicalTrials.gov NCT07820358	Semaglutide, Weight Loss, and LNG-IUD for Conservative Treatment of Endometrial Atypical Hyperplasia and Grade 1 Endometrial Cancer	United States	Grade 1 endometrioid endometrial cancer; EAH/EIN also eligible	Open-label, single-arm interventional study	Semaglutide plus LNG-IUD and structured weight loss	No concurrent control arm	Pathological response rate at 6 months	Not yet recruiting	2031-12-31 (Estimated)	No results posted
ClinicalTrials.gov NCT07812636	Tirzepatide and Levonorgestrel IUD Therapy for EIN and Early-Stage Endometrial Cancer	United States	Grade 1 endometrioid endometrial carcinoma; EIN also eligible	Open-label, single-arm pilot study	Tirzepatide plus LNG-IUD	No concurrent control arm	Pathological response at approximately 6 months	Not yet recruiting	2028-08-01 (Estimated)	No results posted
ClinicalTrials.gov NCT07349641	A Study of Weight Loss Intervention With Tirzepatide and Progestin Intrauterine Device to Treat Endometrial Hyperplasia and Grade 1 Endometrial Cancer	United States	Grade 1 endometrial cancer; AH/EIN also eligible	Phase II, open-label, single-arm study with historical controls	Tirzepatide plus LNG-IUD	Historical LNG-IUD-only controls	Weighted pathological complete response at 26 weeks	Not yet recruiting	2030-10-31 (Estimated)	No results posted
ClinicalTrials.gov NCT07078838	Tirzepatide in Women With Obesity and Endometrial Intra-epithelial Neoplasia or Grade 1 Endometrial Cancer	United States	Grade 1 endometrial cancer; EIN also eligible	Randomized, open-label, parallel-group preoperative study	Tirzepatide	Standard care without tirzepatide	Antiproliferative effect assessed by Ki-67 staining at surgery, after 4 weeks	Not yet recruiting	2029-03 (Estimated)	No results posted
ClinicalTrials.gov NCT07065552	Tirzepatide in Obesity-Driven Endometrial Cancer	United States	Endometrial cancer	Open-label, single-arm preoperative window study	Tirzepatide	No concurrent control arm; paired pre/post-treatment tissue	Change in tumour proliferation by Ki-67 staining from baseline to hysterectomy, up to 6 weeks	Recruiting	2028-07 (Estimated)	No results posted
ClinicalTrials.gov NCT06073184	Weight-loss Drug for Fertility-Sparing Treatment of Atypical Hyperplasia and Low-grade Cancer of the Endometrium	Canada	Low-grade endometrial cancer; atypical hyperplasia also eligible	Open-label, single-arm interventional study	Semaglutide plus progestin IUD	No concurrent control arm	Complete pathological response at 48 weeks	Recruiting	2032-02 (Estimated)	No results posted
ClinicalTrials.gov NCT06751589	A Study of a Weight Loss Intervention in People With Endometrial Cancer	United States	Endometrial cancer during chemotherapy	Open-label, single-arm interventional study	Tirzepatide or semaglutide	No concurrent control arm	Treatment completion: at least 70% of weekly doses (17/24); assessment at 1 year	Recruiting	2027-12 (Estimated)	No results posted
ClinicalTrials.gov NCT07462663	SHAPE-ENDO: Pilot Randomized Trial of Multimodal Pre-Surgical Optimization Versus Standard Surgery in Patients With Obesity and Early-Stage Endometrial Cancer	Spain	Low-risk endometrioid endometrial cancer; AH/EIN also eligible	Randomized, open-label, parallel-group pilot study	Semaglutide within a multimodal strategy: progestin therapy, nutrition and exercise	Standard immediate surgery	Recruitment, randomization acceptance, retention, adherence and strategy completion; surgery without progression; serious adverse events, progression and discontinuation	Not yet recruiting	2035-01 (Estimated)	No results posted
ClinicalTrials.gov NCT07605247	Tirzepatide in the Treatment of Endometrial Cancer	NR	Endometrial cancer; precancerous lesions also described	Phase II pilot; inconsistent design information [a]	Tirzepatide plus LNG-IUD	LNG-IUD alone, as described in the summary [a]	Quality of life through patient-reported outcomes at screening and 3, 6 and 9 months [a]	Not yet recruiting	2028-01 (Estimated)	No results posted
Clinical Trials Information System CTIS2025-523877-40-00	A clinical trial studying the effect of Tirzepatide in women living with endometrial cancer	Ireland	Grade 1–2 endometrioid endometrial adenocarcinoma	Phase II; randomization referenced, but allocation fields are inconsistent [b]	Tirzepatide plus LNG-IUS	NR	Pathological complete response at 12 months	Not Recruiting	2029-04-19 (Estimated)	NR
ChiCTR ChiCTR2500102009	The role of tirzepatide in fertility-sparing treatment in obese and overweight patients with endometrial cancer and atypical hyperplasia	China	Endometrial cancer; atypical hyperplasia also eligible	Single-arm interventional study	Tirzepatide plus standard fertility-preserving treatment	No concurrent control arm	Percentage weight reduction; reversal of endometrial lesions; time to reversal	Not Recruiting	Not found	NR
ChiCTR ChiCTR2600129728	A multicenter real-world study of metabolic regulation combined with progesterone for the care and treatment of obese patients with endometrial cancer and atypical endometrial hyperplasia	China	FIGO IA, grade 1 endometrial adenocarcinoma; atypical hyperplasia also eligible	Observational cohort study	Semaglutide plus progestin	Metformin plus progestogen	Pathological complete remission at 3 months and within 12 months	Not Recruiting	Not found	NR

REGISTRY / TRIAL ID	STUDY TITLE	COUNTRY / REGION	CANCER TYPE	STUDY DESIGN	GLP-1–BASED INTERVENTION	COMPARATOR	PRIMARY ENDPOINT(S)	RECRUITMENT STATUS	ESTIMATED/ACTUAL COMPLETION DATE	RESULTS AVAILABLE
ClinicalTrials.gov NCT06877572	Weight Loss Management in Endometrial Cancer Survivors	United States	Early-stage endometrial cancer survivors	Prospective observational cohort	Weight management pharmacotherapy, including GLP-1 agonists; other drug classes also offered	Weight management without pharmacotherapy	Proportion of referred participants starting weight management medication within 12 months	Active, not recruiting	2030-10 (Estimated)	No results posted
ClinicalTrials.gov NCT07676331 CTIS 2026-525820-76-00	Clinical Study of Local and Systemic Biological Impact of GLP1/GIP-Receptor Agonists in Patients With Breast Cancer: The CLARA Trial	Belgium	HR-positive, HER2-negative early breast cancer	Phase II, randomized, open-label, parallel-group study (ClinicalTrials.gov) [c]	Tirzepatide, alone or with letrozole	Immediate surgery and letrozole comparison arms	Change in log-transformed Ki-67 expression from baseline to surgery, approximately 4–5 weeks	Not yet recruiting	2028-05-01 (Estimated)	No results posted
ClinicalTrials.gov NCT06517212	Tirzepatide Weight Loss for MRD+ Early Breast Cancer	United States	HR-positive, HER2-negative, node-positive early breast cancer; MRD-positive	Open-label, single-arm interventional study	Tirzepatide	No concurrent control arm	Plasma ctDNA clearance within 2 years; proportion alive and free of distant metastatic disease at 2 years	Recruiting	2030-12-31 (Estimated)	No results posted
ClinicalTrials.gov NCT06518837	Tirzepatide for Weight Loss Intervention in Early-Stage Hormone Receptor Positive/HER2 Negative Breast Cancer	United States	HR-positive, HER2-negative early breast cancer during adjuvant treatment	Open-label, single-arm interventional study	Tirzepatide	No concurrent control arm	At least 5% weight loss during adjuvant treatment; study duration up to 2 years	Recruiting	2027-09-30 (Estimated)	No results posted
ClinicalTrials.gov NCT07257484	Tirzepatide's Role in Postmenopausal HR+ Breast Cancer Survivors	United States	Postmenopausal HR-positive breast cancer survivors	Open-label, single-arm interventional study	Tirzepatide	No concurrent control arm	Proportion completing the week 24 visit with all required assessments	Recruiting	2028-02-25 (Estimated)	No results posted
ClinicalTrials.gov NCT07769047	Semaglutide and Structured Lifestyle Support to Improve Heart and Blood Vessel Health in Postmenopausal Obese Breast Cancer Survivors Taking Aromatase Inhibitors	United States	Stage I–III breast cancer survivors taking aromatase inhibitors	Phase IV, randomized, parallel-group study; outcome-assessor masked	Semaglutide plus structured lifestyle support	NR	Change in pulse wave velocity; change in protein expression; enriched biological pathways and gene sets among differentially expressed proteins, up to 24 weeks	Not yet recruiting	2035-07-30 (Estimated)	No results posted
ClinicalTrials.gov NCT07727473	Preoperative Semaglutide Prior to Radical Prostatectomy	United States	Intermediate-risk prostate cancer	Nonrandomized, open-label, single-arm exploratory preoperative study	Semaglutide	No concurrent control arm; paired biopsy/prostatectomy tissue	Changes in caspase-3 activation, Ki-67, Gleason score and Decipher genomic score from biopsy to prostatectomy, up to 6 months	Not yet recruiting	2029-05 (Estimated)	No results posted
ClinicalTrials.gov NCT06759701	Pre-Surgical Tirzepatide-Assisted Weight Loss in Overweight and Obese Men With Intermediate Risk Prostate Cancer: A Pilot Feasibility Study	United States	Intermediate-risk prostate cancer	Open-label, single-arm pilot feasibility study	Tirzepatide-assisted weight loss	No concurrent control arm	Adverse events graded using CTCAE v5.0; through study completion, approximately 1 year	Recruiting	2028-12-31 (Estimated)	No results posted
ClinicalTrials.gov NCT06908694	Investigation of GLP1-Receptor Agonists in Men With Prostate Cancer Taking Androgen Deprivation Therapy	Canada	Prostate cancer during ADT	Open-label, single-arm interventional study	Semaglutide	No concurrent control arm	Serious adverse events and discontinuation; weight, waist, blood pressure, HbA1c, lipids, PSA, creatinine/eGFR and clinical events; up to 12 months	Active, not recruiting	2027-10-31 (Estimated)	No results posted
ClinicalTrials.gov NCT07202247	Metabolic Interventions (Time-Restricted Eating, GLP1 Receptor Agonist, and Heart Healthy Diet) to Improve Cardiometabolic Health in Prostate Cancer Patients During Androgen Deprivation Therapy, IMPACT-ADT Trial	United States	Prostate cancer during ADT	Phase II, randomized, open-label, parallel-group study	Semaglutide or tirzepatide	Heart-healthy diet; additional time-restricted eating arm	Change in AHA PREVENT 10-year cardiovascular risk at completion of 6 months of ADT and 12 months after ADT	Recruiting	2028-04-09 (Estimated)	No results posted
ClinicalTrials.gov NCT07314528	Evaluating the Impact of GLP-1 Receptor Agonists With Total Neoadjuvant Therapy in Rectal Cancer	NR	Locally advanced rectal cancer	Randomized, open-label, parallel-group study	GLP-1 RA (semaglutide described) plus TNT	TNT alone	Weight loss; tissue metabolic profiles; inflammatory mediators; mitochondrial fitness; systemic inflammatory profiles; circulating and tumour-resident immune profiles, within 6–12 months	Not yet recruiting	2028-09 (Estimated)	No results posted

REGISTRY / TRIAL ID	STUDY TITLE	COUNTRY / REGION	CANCER TYPE	STUDY DESIGN	GLP-1–BASED INTERVENTION	COMPARATOR	PRIMARY ENDPOINT(S)	RECRUITMENT STATUS	ESTIMATED/ACTUAL COMPLETION DATE	RESULTS AVAILABLE
ANZCTR ACTRN12626000453381	INtentional PreOperative WEight loss for patients with obesity diagnosed with Resectable GastroIntestinal cancer (INPOWER-GI)	Australia	Resectable gastrointestinal cancers	Randomized, blinded, parallel-group trial; phase II/III	Tirzepatide before surgery	NR	Total body weight loss from baseline to preoperative assessment; weight measurements also scheduled postoperatively	Not Recruiting	2028-01-24 (Estimated)	NR
ClinicalTrials.gov NCT07627191	GLP-1 RA Plus SOC Treatment in First-line, Metastatic Pancreatic, Colorectal, or Hepatocellular Cancer	United States	Metastatic pancreatic, colorectal or hepatocellular cancer	Nonrandomized, open-label, parallel-assignment study	Semaglutide with standard first-line anticancer treatment	NR	Grade ≥3 adverse events attributed to GLP-1 RA or its interaction with chemotherapy, up to 6 months or progression	Recruiting	2028-06-30 (Estimated)	No results posted
JPRN JPRN-jRCT1031250836	Safety of tirzepatide for Hcc In patients with Existing steatotic Liver Disease and obesity after curative treatment	NR	Hepatocellular carcinoma after curative treatment	Phase II, open-label, uncontrolled, single-arm study	Tirzepatide for 24 weeks	No concurrent control arm	Incidence of serious acute pancreatitis regardless of causality	Recruiting	2029-11-30 (Estimated)	NR
ClinicalTrials.gov NCT07782359	Impact of Using a GLP1 on Treatment Related Side Effects of Lung Cancer Patients Receiving Lorlatinib	United States	NSCLC during lorlatinib treatment	Open-label, single-arm interventional study	Tirzepatide with lorlatinib	No concurrent control arm	Changes in body weight, lipids, lorlatinib Cmax and Tmax, and bioavailability by partial AUC, at 1 year	Not yet recruiting	2030-06 (Estimated)	No results posted
EU Clinical Trials Register EUCTR2022-003275-42-NL NL-OMON NL-OMON54011	The influence of different DIets on ALECTinib exposure in lung cancer patients;the DIALECT study.	Netherlands	Metastatic NSCLC during alectinib treatment	Nonrandomized crossover study; phase IV	Semaglutide with alectinib	Alectinib without semaglutide; dietary/timing conditions also evaluated	Alectinib trough concentration under dietary/timing conditions and with/without semaglutide	Completed	2023-12-19 (Actual)	NR
JPRN JPRN-jRCT1031200022	The effect and safety of dulaglutide for hyperglycemia associated with R-CHOP thepapy	NR	Malignant lymphoma during R-CHOP treatment	Phase II, open-label, uncontrolled, single-arm study	Dulaglutide	No concurrent control arm	Rate of change in HbA1c from baseline to months 1–6	Recruiting	2023-06-30 (Estimated)	NR
ClinicalTrials.gov NCT07228741	Resistance Exercise and Incretin Mimetic for Cardiometabolic Health in Survivors of ALL With Obesity	United States	Adult survivors of childhood ALL	Phase II, open-label, single-arm interventional study	Tirzepatide plus supervised resistance exercise	No concurrent control arm	Proportion of evaluable participants achieving at least 5% weight loss at week 28	Recruiting	2028-11 (Estimated)	No results posted
ClinicalTrials.gov NCT07513259	Pre-Diagnosis GLP-1 Receptor Agonist Use and Post-Cancer Mortality in Adults With Obesity and Type 2 Diabetes	United States (TriNetX data); Taiwan (study site)	Obesity-associated cancers	Retrospective observational cohort	GLP-1 RA use before cancer diagnosis; individual agents NR	SGLT2 inhibitors, DPP-4 inhibitors, sulfonylureas, metformin and usual care	All-cause mortality within 36 months after cancer diagnosis in five comparator analyses	Completed	2025-10-31 (Actual)	Results posted 17 August 2026
